# Comparative Effectiveness of Behavioural Sodium-Reduction Interventions for Intensive Systolic Blood Pressure Control in Populations with Elevated Blood Pressure: A Systematic Review and Network Meta-Analysis

**DOI:** 10.3390/nu18030428

**Published:** 2026-01-28

**Authors:** Prapichaya Prommas, Manae Uchibori, Santosh Kumar Rauniyar, Shuhei Nomura

**Affiliations:** 1 Department of Health Policy and Management, Keio University School of Medicine, 35 Shinanomachi, Shinjuku-ku, Tokyo 160-8582, Japan; 2 Ocean Policy Research Institute, Sasakawa Peace Foundation, 1-15-16 Toranomon, Minato-ku, Tokyo 105-8524, Japan; 3 Global Health Policy Lab, International Research Institute of Disaster Science (IRIDeS), Tohoku University, 468-1 Aoba, Aramaki, Aoba-ku, Sendai 980-8572, Miyagi, Japan; 4 Department of Food, Nutrition and Health, Graduate School of Public Health, International University of Health and Welfare, 4-1-26 Akasaka, Minato-ku, Tokyo 107-8402, Japan; 5 Global Research Institute, Keio University, 2-15-45 Mita, Minato-ku, Tokyo 108-8345, Japan

**Keywords:** sodium reduction, salt substitutes, blood pressure, hypertension, network meta-analysis, randomised controlled trials

## Abstract

**Background:** Globally, an estimated 1.4 billion people had hypertension in 2014, yet only just over 20% had controlled blood pressure, and about 580 million remained undiagnosed. Evidence indicates that salt substitutes facilitate meaningful blood-pressure reductions, yet their implementation remains restricted by social and healthcare constraints. The comparative effectiveness of alternative sodium-reduction interventions for elevated blood pressure remains unclear, limiting their introduction across diverse clinical and public health contexts. This study is registered with PROSPERO (CRD420251130153). **Methods:** We systematically searched PubMed, MEDLINE, and supplementary sources for randomised controlled trials (RCTs) published between 2000 and 2025. All behavioural sodium-reduction interventions among populations with elevated blood pressure, including hypertension, were included. The mean difference in systolic blood pressure (SBP) was the primary outcome, as evidence indicates that intensive control of SBP to levels below 120–130 mmHg is significantly associated with a reduced risk of major cardiovascular disease (CVD) and all-cause mortality. Network and subgroup pairwise meta-analyses were performed, with sensitivity analyses conducted to assess robustness of the findings and subgroup analyses used to explore clinical and public health factors influencing intervention effectiveness (clinical factors: blood pressure stage, trial duration, and medication status; public health factors: setting, implementation period, and country income level). **Results:** Of 10,404 records identified, 42 studies (46 trials, *n* = 46,771) were included. While the use of salt substitutes was ranked the most effective intervention in the network meta-analysis, with reductions of −6.78 mmHg (95% CI, −8.42, −5.14) compared to no intervention and −5.35 mmHg (95% CI, −7.89, −2.81) compared to conventional health education, self-monitoring devices and low-sodium diets, when combined with health education, demonstrated similar magnitudes of SBP reductions. Digital health education showed a larger point estimate for SBP reduction by −3.59 mmHg (95% CI −7.40 to 0.22) than conventional education (−1.43 mmHg; 95% CI −3.49 to 0.63), but both confidence intervals crossed zero, indicating no statistically significant difference. Subgroup analyses indicated that, except for trial duration, intervention setting, and country income level in specific intervention comparisons, clinical and public health factors did not generally account for differences in SBP reduction. No evidence of publication bias was observed, except between salt substitutes and no intervention and low-sodium diets and no intervention. **Conclusions:** Network meta-analysis ranked the use of salt substitutes as the most effective intervention, yet self-regulated interventions, such as low-sodium diets and self-monitoring devices, when combined with education-based sodium-reduction approaches, showed comparable point estimates for SBP reductions. Digital health education showed promise as a supportive adjunct to self-regulated interventions, although its effects were variable and require further quantification. These findings underscore the need for alternative sodium-reduction interventions supported by digital or conventional health education to improve blood pressure control. Health education on sodium reduction, including clinical counselling, should be viewed primarily as a complementary component that enhances other interventions.

## 1. Introduction

High sodium intake is a primary factor contributing to the development of hypertension [1], which demonstrates the strongest association with cardiovascular diseases (CVDs) [2]. In 2021, the high consumption of diets containing excess sodium accounted for 4.13 million disability-adjusted life years (DALYs), mainly due to cardiovascular diseases (CVDs), and 1.86 million deaths worldwide [3]. Individuals with high sodium consumption exhibited a 19% higher adjusted rate of cardiovascular disease compared to those with low sodium intake [4]. This association is particularly concerning in light of the estimated 1.4 billion individuals affected by hypertension in 2014, of whom slightly more than 20% had controlled blood pressure, with the majority left untreated [5]. Furthermore, approximately 580 million individuals with hypertension remain unaware of their condition due to a lack of diagnosis [6]. Given the substantial burden of untreated populations with elevated blood pressure worldwide, decreasing dietary sodium consumption helps lower blood pressure and reduces the risk of hypertension [7]. Intensive control of systolic blood pressure (SBP) to below 120–130 mmHg is significantly associated with a reduced risk of major CVD and all-cause mortality [8,9].

Sodium reduction is, therefore, fundamental to achieving the systolic blood pressure (SBP) targets associated with improved cardiovascular outcomes. The World Health Organization (WHO) recommends a sodium intake of less than 2000 mg/day, equivalent to less than 5 g/day or approximately one teaspoon of salt, whereas the average global intake in 2019 was nearly double at 4310 mg/day [1,10]. For individuals with hypertension, the WHO recommends a more stringent sodium intake target of less than 1500 mg/day [11,12]. This recommendation is reinforced by evidence demonstrating a clear linear dose–response relationship between sodium reduction and blood pressure in study groups with a mean blood pressure above the 75th percentile (131/78 mmHg) [13]. Specifically, a reduction of 100 mmol of sodium per day (≈2300 mg/day) was associated with a mean SBP decrease of 7.7 mmHg. Given this dose–response relationship, the recent WHO guidelines recommend the use of lower-sodium salt substitutes (LSSS) as alternatives to regular sodium chloride (NaCl) salt to further support blood pressure reduction [14]. Related evidence from systematic reviews and meta-analyses demonstrated the effects of salt substitutes combined with health education on nutrition, which contributed to a significant reduction in SBP by 7.44 mmHg across populations with and without hypertension [15]. The use of salt substitutes without health education resulted in a reduction of 4.9 mmHg among both groups [16], and among hypertensive populations, a decrease in SBP by 4.61 mmHg was achieved with salt substitutes [17]. Nevertheless, despite their significant benefits in reducing blood pressure, there are ongoing safety concerns about the use of LSSS containing potassium chloride (KCl), particularly for individuals with kidney dysfunction [14,18].

Despite the demonstrated effectiveness of alternative formulations, salt substitutes may be challenging to implement as a large-scale sodium-reduction intervention due to prevailing dietary patterns and the current state of the food industry. In many countries, approximately 70–80% of sodium intake is reported to occur via commercial prepared foods rather than home cooking [19,20]. In the United States, salt-substitute use consistently remained below 5.6% among both untreated hypertensive and normotensive adults throughout 2003–2020 [21]. Although a systematic review indicates that mandatory reformulation may be more effective than voluntary measures [22], implementation across EU member states has not yet achieved recommended sodium-intake targets, largely due to food-safety concerns surrounding sodium alternatives and challenges with consumer acceptability [23].

Meanwhile, evidence from systematic reviews and meta-analyses indicates that self-regulated behavioural change interventions have been shown to effectively lower sodium intake, in part due to reduced discretionary use and improved purchasing decisions [24]. However, the magnitude of SBP reductions reported in these pairwise meta-analyses was considerably smaller than that observed with the implementation of salt substitutes. One study investigating the DASH (Dietary Approaches to Stop Hypertension) diet with low sodium reported a decrease in SBP of 5.20 mmHg in both hypertensive and non-hypertensive groups [25]. Furthermore, another study found that using self-monitoring devices to measure the urinary sodium-to-potassium ratio decreased SBP by 2.45 mmHg in both groups [26]. In parallel, evidence from a systematic review showed that nutrition education interventions, whether delivered independently or alongside other interventions, were associated with reductions in sodium intake [27]. Consistent with this finding, another meta-analysis indicated that a multi-behaviour intervention, combining salt-restriction spoons, self-monitoring devices for urinary sodium, and health education and cooking classes on sodium reduction, reduced SBP by 1.17 mmHg [28].

Despite extensive synthesis through systematic reviews and pairwise meta-analyses, there is limited evidence concerning (1) the comparative effectiveness of all sodium-reduction interventions on blood pressure control, quantifying differential SBP reductions across interventions; (2) the effects on populations with elevated blood pressure, whether treated or untreated; and (3) comprehensive evaluations of clinical and public health factors that influence intervention effectiveness, which have not been thoroughly investigated. Recent evidence from a systematic review and meta-analysis reported no significant differences in SBP reduction across behavioural sodium-reduction interventions [29]. This underscores the need for an integrated analytical approach in which network meta-analysis, complemented by subgroup pairwise analyses, enables a comprehensive comparison of all available sodium-reduction interventions. Pairwise meta-analyses alone cannot evaluate all interventions concurrently, particularly when direct head-to-head trials are lacking. Network meta-analysis fills this gap by enabling simultaneous comparisons and producing a coherent ranking of treatment effectiveness. This approach also facilitates effective control of SBP by addressing individual behavioural and practical constraints while offering evidence-based alternatives for broader population implementation. The main objective of this study was to assess the comparative effectiveness of behavioural sodium-reduction interventions for reducing SBP in populations with elevated blood pressure, using evidence from randomised controlled trials (RCTs) published over the past 25 years. Two research questions were addressed:

Q1. What is the comparative effectiveness of behavioural sodium-reduction interventions on SBP reduction relative to conventional health education on sodium reduction and no intervention?

Q2. Which clinical and public health factors influence the effectiveness of these interventions?

## 2. Methods

### 2.1. Search Strategy and Selection Criteria

Our systematic review and meta-analysis were conducted in accordance with the PRISMA extension guideline for systematic reviews incorporating network meta-analyses for health interventions [30]. We searched two electronic databases (PubMed and Medline) from 1 January 2000 to 28 February 2025, spanning 25 years. The search strategy consisted of three sets of keywords: (1) salt-reduction-related terms (for example, “salt reduction”, “sodium reduction”, “salt intake”, “sodium intake”, “salt consumption”, “sodium consumption”), (2) elevated blood pressure-related terms (for example, “hypertension” OR “high blood pressure” OR “systolic blood pressure” OR “diastolic blood pressure” OR “hypertensive disorder”), and (3) research-method-related terms (for example, “trial”, “randomised controlled trial”). To include all types of interventions, we did not use intervention-related keywords in the search strategy. The details of the search strategy are provided in the Supplementary Materials (Supplementary Table S1). We also examined the reference lists of the included studies from previous systematic reviews, Google Scholar, and leading organization websites, including those of the WHO, to ensure comprehensive and accurate study inclusion.

The complete inclusion and exclusion criteria are presented in Supplementary Table S2. Eligible studies were primary studies with randomised controlled trials (RCTs), including multi-arm designs, that compared predefined interventions with appropriate comparator groups, such as no intervention or usual care, or conventional health education; in real-world or experimental settings; written in English; published between 1 January 2000, and 28 February 2025; and provided full-text access or abstracts with sufficient outcome information and key trial characteristics. The participants were adults aged ≥18 years, in accordance with hypertension screening guidelines [31], with SBP ≥ 120 mmHg (elevated blood pressure) or ≥140 mmHg [32] (clinically diagnosed or self-reported elevated blood pressure and/or hypertension). The primary outcome was the mean difference (MD) in SBP between intervention and control groups among populations with elevated blood pressure, including those with hypertension. We excluded non-randomised controlled studies without control groups, as well as commentaries, opinion pieces, and systematic reviews. We also excluded studies reporting RCTs published outside the predefined inclusion period; articles written in languages other than English; and studies with abstracts lacking sufficient outcome information. In addition, studies involving participants aged <18 years or adults with SBP < 120 mmHg were excluded. Studies reporting outcomes not comparable with our primary endpoint, such as dietary sodium intake or within-group SBP changes without a comparator arm, were also excluded.

### 2.2. Data Extraction

Using Rayyan QCRI, a web-based tool for systematic reviews (http://www.rayyan.ai/), two reviewers (PP and RS or PP and MM) independently assessed the identified records for eligibility by title, abstract, and full text. Differences in opinion were resolved through consensus with the project leader, SN. Extracted information was collected in a standardized form (Supplementary Table S3) listing the study ID, the first author’s last name, the publication year, the study country, the studied country’s income level, the sample size, the sample’s mean age, the percentage of female participants, the included SBP level, the intervention treatment name, the control treatment name, the number of participants in the intervention and control groups, the period of study (before or after 13 May 2013, when the WHO announced a target 30% reduction in global sodium intake by 30% [33]), and the trial duration. Detailed information, including the mean difference in SBP (mmHg) and its standard error (SE), was also recorded. All treatment arms from multi-arm trials were incorporated intact into the network meta-analysis, with correlations appropriately accounted for to avoid splitting shared comparator groups [34]. This approach maintains the statistical dependency of the intervention arms within the same studies, thereby preventing biased estimates. In pairwise meta-analyses, shared control groups were allocated proportionally to prevent double counting [35]. This method ensures that each participant is included only once in the pooled analysis and that weighting remains balanced across all comparisons. Classification of interventions is based on the primary intervention components. For multi-component interventions, studies were assigned according to dominant components, as defined by study authors. Two reviewers independently double-checked the extracted data and intervention classifications. If a study demonstrated multiple estimates from stratified analysis (for example, by sex or stage of result assessment), these estimates were aggregated to provide a single mean difference (MD). For studies with missing information, the term “not defined” (N/D) was recorded in the template.

### 2.3. Study Quality Assessment

Given the potential value of studies published over the 25-year period, we assessed the quality of the studies included after the final selection. To evaluate study quality, we used the Joanna Briggs Institute (JBI) checklist for randomised control trials (RCTs) [36]. Two reviewers (PP and either RS or MM) independently appraised the quality of the studies before reaching consensus. Disagreements were resolved by a third reviewer (either R.S., M.M., or S.N.). The total score, ranging from 0 to 13, was calculated as the sum of “yes” responses. Quality was categorized as follows: scores of 1–4 indicated low quality, 5–8 indicated medium quality, and 9–13 indicated high quality [37]. Studies lacking full texts received a “not applicable” quality rating.

### 2.4. Risk of Bias Assessment

In addition to excluding low-quality trials identified through the quality assessment, we conducted sensitivity analyses that removed highly influential studies or outliers, identified through funnel-plot inspection from the network meta-analysis, to assess the robustness of the findings. To examine sources of heterogeneity, we also performed subgroup analyses, with *p*-values < 0.05 indicating statistically significant subgroup effects [38]. Given the limited number of trials, outliers were excluded only when statistically significant subgroup differences were identified. We aimed to include “intention-to-treat (ITT)” trials when data were available. Due to the limited number of trials, we also excluded per-protocol (PP) outcomes when statistically significant subgroup differences were observed between ITT and PP trials. In cases where outlying trials exerted a substantial influence on heterogeneity from statistically significant subgroup differences or a tendency toward difference with outliers between ITT and PP trials was observed when comparing analyses with and without outliers, an additional network meta-analysis excluding these trials was conducted to assess the robustness and consistency of the results. We also compared the network meta-analysis results with corresponding pairwise meta-analyses to verify the consistency of effect estimates. When publication biases were detected, Egger’s regression test was conducted, with a *p*-value of less than 0.05 indicating significant publication bias [35]. These conclusions were further supported by examining funnel-plot asymmetry when the meta-analysis included at least ten studies [39].

### 2.5. Statistical Analyses

The analytical and synthesis procedure was conducted in two stages: (stage 1) comparison of effects using network meta-analysis and (stage 2) analysis of clinical and public health factors using subgroup pairwise meta-analyses.

Stage 1: Comparison of effects using network meta-analysis

We conducted a network meta-analysis (NMA) within the random-effects framework to combine both direct and indirect intervention effects into a single pooled estimate [40]. The Netmeta package in the R library (version 4.3.2) was employed to conduct the network meta-analysis (NMD) (Supplementary Table S4). In the NMA, we used the *p*-score to rank all interventions in a rank plot [41]. A higher *p*-score indicates greater confidence that one intervention is more effective than the others. A minimum of two studies for intervention comparison were required for inclusion in the analysis [42]. In addition to comparing the effects of all interventions in the NMA, we used a forest plot to conduct comparative analyses of selected interventions against chosen comparators, whether those comparisons were direct (head-to-head) or indirect across studies. The selected comparators were “conventional health education” and “no intervention”.

To assess network consistency, we evaluated direct and indirect evidence using both local and global approaches. Local inconsistency was examined using node-splitting models, while global inconsistency was assessed using the design-by-treatment interaction model. No statistically significant inconsistency was detected (*p* > 0.05), indicating good agreement between direct and indirect estimates. According to the predefined eligibility criteria, no missing outcome data relevant to the network meta-analysis were identified among the included studies. Therefore, no imputation procedures were required.

Stage 2: Analysis of clinical and public health factors using subgroup pairwise meta-analyses

We performed pairwise meta-analyses using RevMan to analyse subgroup differences based on (1) clinical factors, including (1.1) blood pressure stage (elevated BP and hypertension diagnosed or self-reported), (1.2) trial duration (≤3 months, >3 months to 6 months, >6 months to 12 months, and >12 months), (1.3) medication status during trails (yes and no), and (1.4) type of trials (intention-to-treat and per-protocol), and (2) public health factors, including (2.1) settings (clinical, community, or care home), (2.2) study period (before or after May 2013, following the introduction of the World Health Organization’s sodium-reduction target of 30%) [33], and (2.3) country income level [43]. This subgroup analysis aimed to determine whether individual interventions resulted in statistically significant SBP reductions within each subgroup category. Subgroup differences with *p*-values < 0.05 were regarded as statistically significant subgroup effects [38]. The mean differences in office SBP between interventions and controls were analysed with respect to clinical factors. For public health factors, combined approaches to SBP (office, home, and ambulatory) were analysed because the synthesis was not limited to clinical settings. Both were conducted at post-intervention main time points, using a random-effects model that assumes constant heterogeneity across intervention comparisons. We reported the intervention effect estimates of each subgroup category as mean differences (MDs) with corresponding 95% confidence intervals or standard errors. The mean difference represents the unadjusted change in the outcome between the experimental and comparator groups of each randomized controlled trial [44]. The heterogeneity (*I*^2^) ranged from 0% to 95%, indicating a wide range of heterogeneity in the included trials. The commonly referenced cutoff values of 25%, 50%, and 75% for *I*^2^ indicate minor, moderate, and significant heterogeneity [45]. Outside the formal test for subgroup differences, we restricted the interpretation of individual subgroup effects when substantial within-subgroup heterogeneity was present.

To ensure transitivity, we also assessed subgroup differences to examine the distribution of potential effect modifiers across all treatment comparisons. Assessment of potential effect modifiers demonstrated only minor clinically and public health–relevant imbalances across comparisons, supporting the plausibility of indirect treatment comparisons.

## 3. Results

### 3.1. Study Characteristics

**Figure 1:**
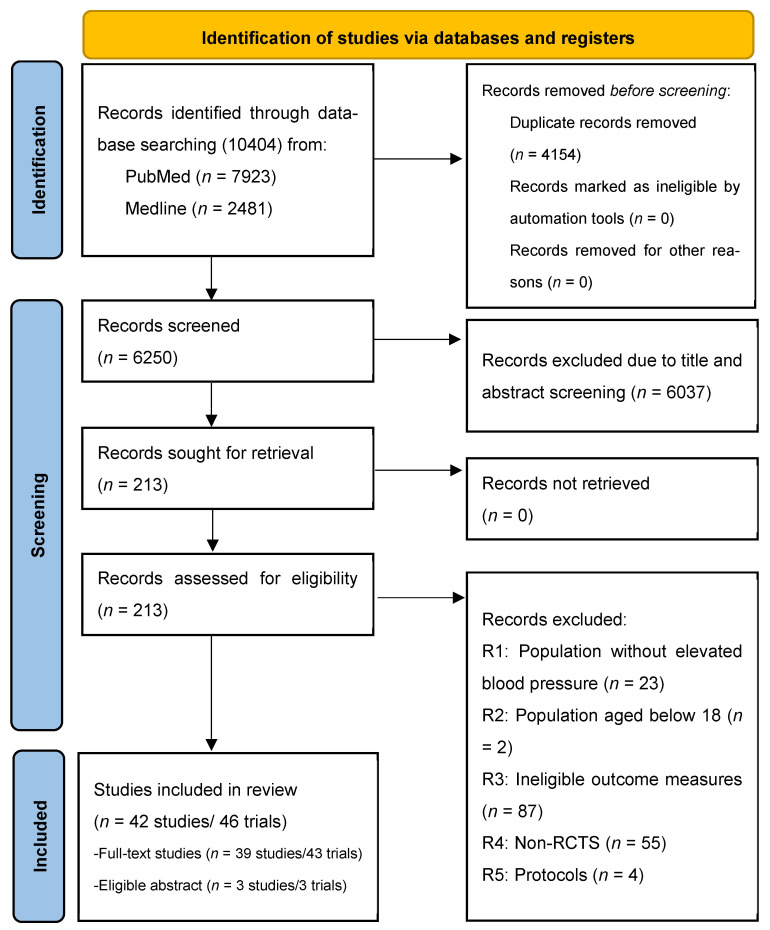
PRISMA flow diagram.

Figure 1 presents the PRISMA flow chart outlining the study selection process. The initial search identified 10,404 records from database searching including PubMed (7923 studies) and Medline (2481 studies). After removing 4154 duplicate records, we screened 6250 records by title and abstract to assess their potential eligibility. After removing 6037 records after title and abstract screening, 213 reports including full-text articles and eligible abstract-only studies containing essential numerical data for effect-size calculation, were reviewed for eligibility. Of these 213 studies, 71 reports were excluded. Specifically, 25 involved ineligible study populations, 87 reported non-comparable outcome measures for mean SBP, 55 were non-randomised controlled trials, and 4 were protocol studies. Ultimately, 42 studies comprising 46 trials were included in the meta-analysis. Of these, 39 studies (43 trials) were full-text publications, and three eligible abstracts contributed three additional trials.

A summary of the 42 included studies, comprising 46 trials (three with two intervention comparisons and one with three intervention comparisons) enrolling 46,771 participants, is presented in Supplementary Table S3. The characteristics of the included studies are presented in Supplementary Table S5. Across included studies, seven non-pharmacological interventions aimed at reducing sodium intake among populations with elevated blood pressure, including individuals with hypertension, were identified in the included studies: (1) salt substitutes; (2) low-sodium diets; (3) low-sodium diets with conventional health education on sodium reduction; (4) self-monitoring devices for urinary salt excretion with conventional health education on sodium reduction, (5) digital health education on sodium reduction, (6) conventional health education on sodium reduction, including counselling and provision of health education materials; and (7) no intervention. The most common trials were comparisons between salt substitutes and no intervention (14 studies, 15 trials), followed by comparisons between low-sodium diets and no intervention (seven studies, seven trials), comparisons between low-sodium diets and conventional health education (six studies, six trials), comparisons between conventional health education and no intervention (five studies, eight trials), the comparisons between digital health education and conventional health education (six studies, six trials), comparisons between self-monitoring devices for urinary salt excretion with conventional health education and conventional health education (two studies, two trials), and comparisons between salt substitutes and conventional health education (two studies, two trials). Most studies were conducted in upper-middle income countries (UMICs) (23 studies), particularly China (15 studies), Iran (three studies), Thailand (two studies), Argentina (one study), Brazil (one study), and Indonesia (one study), followed by high-income countries (HICs) (17 studies), particularly the USA (six studies), the UK (two studies), New Zealand (two studies), France (two studies), Australia (one study), Finland (one study), Italy (one study), Japan (one study), and the Netherlands (one study). Only two studies were conducted in low- or middle-income countries (LMICs): Pakistan (one study) and Tibet, China (one study). Twenty-five studies were conducted after May 2013, when the World Health Organization announced its global sodium reduction target. Sixteen studies were conducted before May 2013, and one study that began before May 2013 was completed after that time. Finally, 20 studies were determined to be of high quality, while 19 studies were found to be of moderate quality, and quality evaluation of 3 studies was not applicable due to limited access to the full text (Supplementary Table S4).

#### 3.1.1. Salt Substitutes

We identified 14 studies (15 trials, *n* = 41,808) that evaluated the effect of “*salt substitutes*” (*n* = 20,982) on SBP compared to “*no intervention*” (*n* = 20,819) [46,47,48,49,50,51,52,53,54,55,56,57,58,59]. Thirteen studies (92.86%, 14 trials) reported office SBP, while one study (7.14%, one trial) reported home SBP [47]. Regarding medication use, four studies (28.57%, four trials) reported no blood pressure medication use [46,52,53,58], one study (7.14%, two trials) reported medication use [55], and nine studies (64.29%, nine trials) did not report medication status [47,48,49,50,51,54,56,57,59]. In terms of blood pressure stage, six studies reported populations with diagnosed hypertension (42.86%, seven trials) [47,48,50,52,54,55], three studies (21.43%, three trials) reported determined hypertension [57,58,59], two studies (14.29%, two trials) reported diagnosed elevated blood pressure [46,49], and three studies (21.57%, three trials) reported determined elevated blood pressure) [51,53,56]. Regarding the trial duration, four studies (28.57%, four trials) reported ≤3 months [46,50,53,58], three studies (21.14%, four trials) reported >6 months to 12 months [47,55,57], and seven studies (57.14%, seven trials) reported >12 months [48,49,51,52,54,56,59].

Among these fourteen studies, two (14.29%, two trials) were conducted in a clinical setting [47,54], ten (71.42%, 11 trials) were conducted in community settings [46,48,49,50,52,53,55,57,58,59], and two (14.29%, two trials) were conducted in care homes [51,56]. Geographically, two studies (14.29%, two trials) were conducted in HICs [46,53], eleven studies (78.57%, 12 trials) in China, a UMIC [47,48,49,50,51,52,54,55,56,58,59], and one study (7.14%, one trial) in an LIC [57]. Regarding the study period, seven studies (50.00%, seven trials) were conducted before May 2013 [47,48,50,53,54,57,58], and seven studies (50.00%, eight trials) were conducted afterward [46,49,51,52,55,56,59]. In terms of analysis method, thirteen studies applied an intention-to-treat (ITT) approach (92.86%, 14 trials) [46,47,48,49,51,52,53,54,55,56,57,58,59], while one study (7.14%, one trial) used per-protocol (PP) analysis [50]. 

In addition, we identified two further studies (two trials, *n* = 75) comparing “*salt substitutes*” (*n* = 39) and “*conventional health education*” (*n* = 36) [60,61]. One study reported office SBP [60], while another reported home SBP [61]. Both studies reported populations with diagnosed hypertension and a trial duration of ≤3 months [60,61]. Both were also conducted in a clinical setting and used ITT analysis [60,61]. One study was conducted in an HIC before May 2013 [60], while the other was conducted in a UMIC after May 2013 [61].

*Low-sodium diets*

We identified seven studies (seven trials, *n* = 575) that assessed the effect of “*low-sodium diets*” (*n* = 275) on SBP reduction compared to “*no intervention*” (*n* = 300) [62,63,64,65,66,67,68]. Among these, four studies (57.14%, four trials) investigated the low-sodium DASH (Dietary Approaches to Stop Hypertension) diet [62,63,67,68], while three (42.86%, three trials) investigated other types of low-sodium diets [64,65,66]. Five studies (71.43%, five trials) reported office SBP [62,64,65,67,68], while two studies (28.57%, two trials) reported 24-h ambulatory SBP [63,66]. Regarding medication use, three studies (42.86%, three trials) reported no blood pressure medication use [62,63,66], three studies (42.86%, three trials) reported stable use [64,65,68], and one study (14.28%, one trial) did not report the status [67]. In terms of blood pressure stage, one study reported populations with diagnosed hypertension (14.29%, one trial) [68], one study reported determined hypertension (14.29%, one trial) [67], two studies (28.57%, two trials) reported diagnosed elevated blood pressure [62,63], and three studies (42.85%, three trials) reported determined elevated blood pressure [64,65,66]. Regarding the trial duration, five studies (71.43%, five trials) reported ≤3 months [64,65,66,67,68], and two studies (28.57%, two trials) reported >3 months to 6 months [62,63].

Among these seven studies, three (42.85%, three trials) were conducted in a clinical setting [62,63,68], while four (57.15%, four trials) were conducted in a community setting [64,65,66], including one in a school [67]. Geographically, three studies (42.85%, three trials) were conducted in the USA, an HIC [63,66,67], while four (57.15%) (four trials) were conducted in UMICs [62,64,65,68]. Regarding the study period, four studies (57.15%, four trials) were conducted before May 2013 [62,63,65,67], while three studies (42.85%, three trials) were conducted after May 2013 [64,66,68]. In terms of analysis method, five studies applied an ITT approach (71.43%, five trials) [62,64,65,66,67], while two studies used PP analysis (28.57%, two trials) [63,68].

In addition, we identified six studies (six trials, *n* = 2906) comparing “*low-sodium diets*” with “*conventional health education*” (*n* = 1490) and “*conventional health education*” (*n* = 1416) [69,70,71,72,73,74]. All studies were conducted in clinical settings, and all (100.00%, six trials) reported office SBP [69,70,71,72,73,74]. Regarding medication use, four studies (66.66%, four trials) reported no blood pressure medication use [69,71,73,74], one study (16.67%, one trial) reported use [72], and one study (16.67%, one trial) did not report the medication status [70]. In terms of blood pressure status, four studies reported populations with diagnosed hypertension (66.67%, four trials) [69,72,73,74], and two studies (33.33%, two trials) reported diagnosed elevated blood pressure [70,71]. Regarding the trial duration, one study (16.67%, one trial) reported ≤3 months [71], two studies (33.33%, two trials) reported >3 months to 6 months [69,73], two studies (33.33%, two trials) reported >6 months to 12 months [70,74], and one study (16.67%, one trial) reported >12 months [72]. Among these six studies, four (57.14%, four trials) were conducted in HICs [69,70,71,74], one (21.43%) was conducted in a UMIC [73], and one (21.43%) was conducted in an LIC [72]. In terms of analysis method, two studies deployed an ITT approach (33.33%, two trials) [69,71], while four studies (66.67%, four trials) used PP analysis [70,72,73,74].

#### 3.1.2. Self-Monitoring Devices for Urinary Salt Excretion

We identified two studies (two trials, *n* = 131) that evaluated the effect of “*self-monitoring devices*” with “*conventional health education*” (*n* = 67) on SBP compared to “*conventional health education*” (*n* = 64) [75,76]. One study (50%, one trial) reported office SBP [76], while another (50%, one trial) reported home SBP [75]. Both studies reported populations with diagnosed hypertension and a trial duration of ≤3 months. One study (50%, one trial) reported stable blood pressure medication use [76], while another (50%, one trial) did not report the medication status [75]. Geographically, one study (50%) was conducted in an HIC before May 2013 [75], while the other (50%) was conducted in a UMIC after May 2013 [76]. Both studies deployed an ITT approach (100%).

#### 3.1.3. Digital Health Education on Sodium Reduction

We identified six studies (six trials, *n* = 635) that evaluated the effect of “*digital health education on sodium reduction*” (*n* = 322) on SBP compared to “*conventional health education*” (*n* = 313) [77,78,79,80,81,82]. Among these, five studies (83.33%, five trials) investigated smartphone applications [77,78,79,81,82], and one study (16.67%, one trial) investigated web-based self-management [80]. Four studies (66.67%, four trials) reported office SBP [78,80,81,82], and two studies (33.33%, two trials) reported home SBP [77,79]. In terms of blood pressure stage, three studies reported populations with diagnosed hypertension (50%, three trials) [77,80,82], two studies reported determined hypertension (33.33%, two trials) [79,81], and one study (16.67%, one trial) reported determined elevated blood pressure [78]. Regarding medication use, one study (16.67%, one trial) reported no blood pressure medication use [79], three studies (50.00%, three trials) reported stable use [77,80,82], and two studies (33.33%, two trials) did not report the medication status [78,81]. Regarding the trial duration, four studies (66.67%, four trials) reported ≤3 months [77,78,79,82], one study (16.665%, one trial) reported >6 months to 12 months [80], and one study (16.67%, one trial) reported >12 months [81].

Among six studies, four (66.67%, four trials) were conducted in clinical settings [77,80,81,82], while two (33.33%, two trials) were conducted in community settings [78,79]. Geographically, five studies (83.33%, five trials) were conducted in HICs [77,78,79,80,82], while one study (16.67%, one trial) was conducted in China, a UMIC [81]. Regarding the study period, all studies (100%) were conducted after May 2013. In terms of the analysis method, four studies deployed an ITT approach (66.67%, four trials) [77,78,79], while two studies employed PP analysis (33.33%, two trials) [80,81,82].

#### 3.1.4. Conventional Health Education on Sodium Reduction

We identified five studies (eight trials, *n* = 647) that investigated the effect of “*conventional health education on salt reduction*” (*n* = 363) on SBP compared to “*no intervention*” (*n* = 284) [83,84,85,86,87]. All studies reported office SBP. Three studies (60.00%, six trials) reported populations with diagnosed hypertension [83,84,85], while two studies (40.00%, two trials) reported high-risk hypertension [86,87]. Regarding medication use, two studies (40.00%, four trials) reported no blood pressure medication use [84,87], one study (20.00%, one trial) reported stable use [83], and two studies (40.00%, three trials) did not report medication status [85,86]. Regarding the trial duration, two studies (40.00%, two trials) reported ≤3 months [83,86], two studies (40.00%, two trials) reported >3 months to 6 months [84,85], and one study (20.00%, one trial) reported >6 months to 12 months [87].

Among these five studies, two (40%, two trials) investigated dietary counselling on sodium reduction by physicians [83,84], while three (60%, six trials) investigated health education training, including self-education [85,86,87]. Three studies (60%, five trials) were conducted in clinical settings [83,84,86], while two (40%, three trials) were conducted in community settings [85,87]. Geographically, two studies (40%, two trials) were conducted in HICs, while three studies (60%, six trials) were conducted in UMICs. Regarding the study period, four studies (80%, seven trials) were conducted after May 2013 [83,84,85,87], while one study (20%, one trial) was conducted before May 2013 [86]. In terms of analysis method, two studies (40%, four trials) used an ITT approach [83,84], while three studies (60%, four trials) used PP analysis [85,86,87].

### 3.2. Main Findings

Q1. What are the comparative effects of behavioural sodium-reduction interventions on SBP reduction relative to conventional health education on sodium reduction and no intervention?

Intervention effectiveness ranking by mean SBP difference

**Figure 2:**
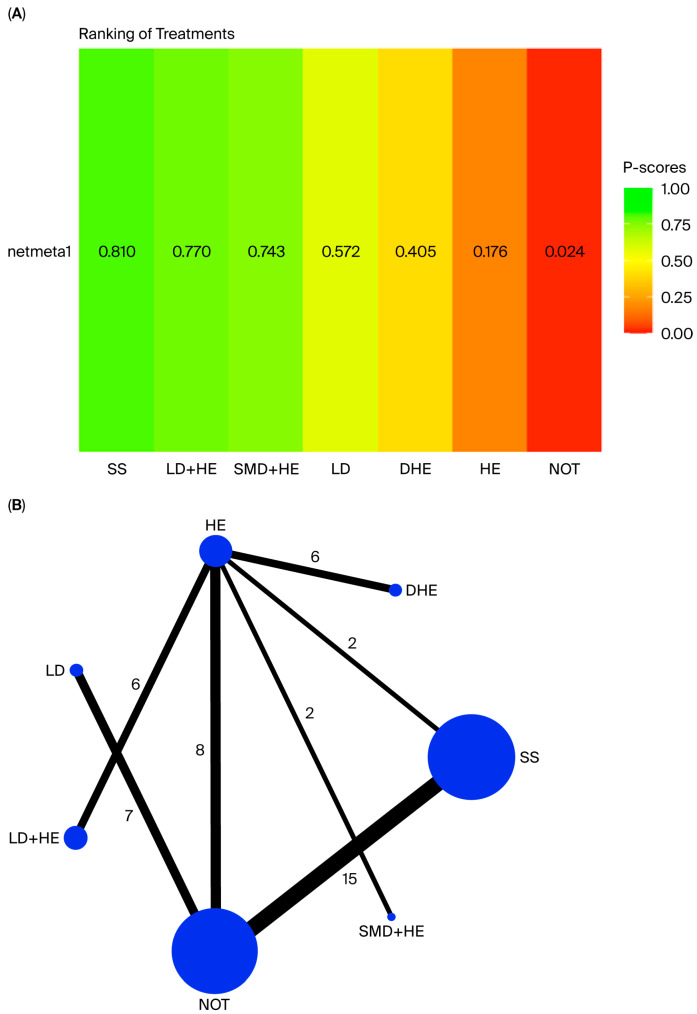
Network meta-analysis diagrams. (**A**) Network ranking plot presenting the effects of the sodium-reduction interventions on SBP control by intervention category, ranked by effect magnitude. (**B**) Network of direct comparison among included interventions. SS (salt substitutes), LD (low-sodium diets), SMD (self-monitoring devices), DHE (digital health education), HE (conventional health education), NOT (no intervention). (**A**) *p*-scores reflect the relative ranking of each intervention in the network meta-analysis, with higher values indicating a greater likelihood of being among the most effective treatments. Salt substitutes (SS) achieved the highest *p*-score (0.810), followed by low-sodium diet plus health education (LD + HE), self-monitoring devices plus health education (SMD + HE), low-sodium diets (LD), digital health education (DHE), conventional health education (HE), and no intervention (NOT). The colour gradient corresponds to the *p*-score scale, ranging from red (least effective) to green (most effective). Each node represents an intervention, with node size proportional to the total number of participants assigned to that intervention across all trials. Edges indicate direct randomized comparisons between interventions, and the thickness of each edge corresponds to the number of contributing trials. Numerical labels on the lines denote the exact number of studies for each pairwise comparison. SS = salt substitutes; HE = conventional health education; NOT = no intervention; LD = low-sodium diets; LD + HE = low-sodium diets plus health education; SMD + HE = self-monitoring devices plus health education; DHE = digital health education.

The *p*-score values from the network meta-analysis rank the interventions based on outcome effectiveness (Figure 2).

The network meta-analysis demonstrated that “*salt substitutes*” was the most effective intervention for reducing SBP among populations with elevated blood pressure (*p*-score of 0.81). This was followed by “*low-sodium diets*” *with* “*conventional health education*”*,* with a *p*-score of 0.77, and “*self-monitoring devices*” with “*conventional health education*”, with a *p*-score of 0.74. Meanwhile, “*digital health education*” and “*conventional health education*” were the least effective interventions, with *p*-scores of 0.405 and 0.176, respectively, among populations with elevated blood pressure.

Comparative mean SBP differences: “*no intervention*” and “*conventional Health education*” as control groups

**Figure 3:**
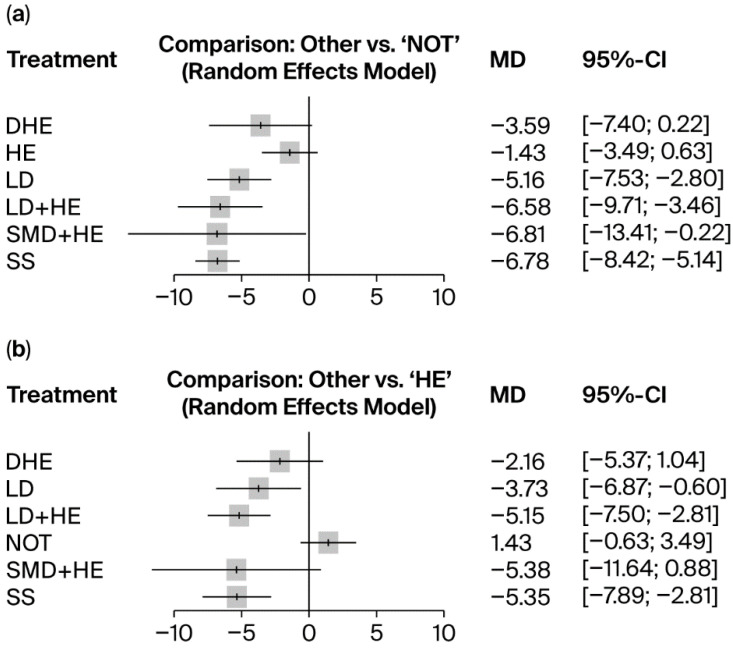
Network Forest plots. (**a**) Mean SBP difference between selected interventions and no intervention (mmHg). (**b**) Mean SBP difference between selected interventions and conventional health education (mmHg). The squares presented pooled estimates of the effect size, and the whiskers demonstrated 95% CI). SS (salt substitutes), LD (low-sodium diets), SMD (self-monitoring devices), DHE (digital health education), HE (conventional health education), NOT (no intervention).

The network meta-analysis forest plots indicate the effect estimates for each intervention compared to a selected control (Figure 3).

Compared to all interventions to “*no intervention*”, “*salt substitutes*” (mean SBP difference, −6.78 mmHg; 95% CI, −8.42, −5.14) and “*self-monitoring devices*” with “*conventional health education*” (mean SBP difference, −6.81 mmHg; 95% CI, −13.41, −0.22) demonstrated the largest reductions in SBP. These were followed by “*low-sodium diets*” *with* “*conventional health education*” (mean SBP difference, −6.58 mmHg; 95% CI, −9.71, −3.46), which also showed a meaningful reduction in SBP. In the absence of concurrent “*conventional health education*”*,* “*low-sodium diets*” alone resulted in a more marginal reduction in SBP (mean SBP difference, −5.16 mmHg; 95% CI, −7.53, −2.80). By contrast, “*Digital health education*” (mean SBP difference, −3.59 mmHg; 95% CI, −7.40, 0.22) and “*Conventional health education*” (mean SBP difference, −1.43 mmHg; 95% CI, −3.49, 0.63) were the two least effective interventions, with confidence intervals crossing the null, indicating greater uncertainty and limited clinical impact.

When compared with “*conventional health education*”, both “*self-monitoring devices*” with “conventional *health education*” (mean SBP difference, −5.38 mmHg; 95% CI, −11.64, 0.88) and “salt substitutes” (mean SBP difference, −5.35 mmHg; 95% CI, −7.89, −2.81) demonstrated the largest reductions in SBP. These were followed by “*low-sodium diets*” with “*conventional health education*” (mean SBP Difference, −5.15 mmHg; 95% CI, −7.50, −2.81), which also showed a meaningful reduction. In contrast, without concurrent “*conventional health education*”, “*low-sodium diets*” produced a smaller reduction in SBP (mean SBP difference, −3.73 mmHg; 95% CI, −6.87, −0.60). “*Digital health education*” presented the least effective intervention (mean SBP Difference, −2.16 mmHg; 95% CI, −5.37, 1.04), with confidence intervals crossing the null, indicating substantial uncertainty.

Q2. What are the clinical and public health factors that impact the effectiveness of the interventions?

Subgroup differences by clinical factors

Subgroup analyses based on clinical factors (blood pressure stage, trial duration, and medication status) showed no statistically significant effect modification across most intervention comparisons (*p* for subgroup > 0.05; Supplementary Table S2), indicating that the overall intervention effects were generally consistent across clinical subgroups.

The only exceptions are a significant difference in trial duration between “*low-sodium diets*” combined with “*conventional health education*” versus “*conventional health education*” (*p* subgroup = 0.01), and between “*conventional health education*” and “*no intervention*” (*p* subgroup = 0.02) (Supplementary Table S8.3). However, given the substantial heterogeneity (*I*^2^ = 72% and 69%, respectively) and the small number of studies per subgroup (1–4 trials), these results should be interpreted with caution [88], as the apparent differences may reflect random variation rather than true effect modification.

Subgroup differences by public health factors

Subgroup analyses based on public health factors (setting, implementation period, and country income level) similarly demonstrated no statistically significant subgroup differences across most comparisons (*p* for subgroup > 0.05; Supplementary Table S7), indicating that the overall intervention effects were generally consistent across public health subgroups.

Statistically significant subgroup differences in country income level were observed between “salt substitute” and “no intervention” (*p* subgroup < 0.05) (Supplementary Table S8.7) and in the intervention setting between “conventional health education” and “no intervention” (*p* subgroup < 0.05) (Supplementary Table S8.5). However, given the substantial heterogeneity (*I*^2^ = 85.9% and 93.9%, respectively) and the small number of studies per subgroup (minimum of three), these results should be interpreted with caution [88], as reflected by the fact that the apparent differences may reflect random variation rather than true effect modification.

### 3.3. Heterogeneity, Publication Bias, and Sensitivity Analysis

There was no evidence of publication bias regarding the pooled mean SBP when comparing the following interventions: “*low-sodium diets*” with “*conventional health education*” and “*conventional health education*” (*p* = 1.00) (Supplementary Figure S1.3), “*Digital health education*” and “*Conventional health education*” (*p* = 0.41) (Supplementary Figure S1.4), and “*conventional health education*” and “*no intervention*” (*p* = 0.74) (Supplementary Figure S1.5). However, evidence of publication bias was found when comparing “*salt substitutes*” and “*no intervention*” (*p* = 0.00) (Supplementary Figure S1.1) and “*low-sodium diets*” and “*no intervention*” (*p* = 0.00) (Supplementary Figure S1.2).

To investigate potential sources of heterogeneity before conducting the network-meta-analysis, sensitivity analyses were conducted. These included the exclusion of influential studies or outliers —those with extreme effect sizes and high variability—as well as PP trials. In the sensitivity analyses (Supplementary Table S9), no significant differences in the findings before and after the exclusion of outliers were identified (*p* subgroup > 0.05). This indicates that excluding outliers does not significantly change the interpretation of the results, except for the “salt substitutes” and “no intervention” comparison. There are also no subgroup differences between ITT and PP trials except for the comparison between “salt substitutes” and “no intervention” (Supplementary Table S8.1). As the “salt substitutes” versus “no intervention” comparison included one PP trial that is an outlier [50], we reassessed the robustness of the original network meta-analysis by conducting an additional sensitivity network-meta-analysis excluding four outliers [48,49,50,54]. Following the exclusion of these four outliers, the ranking remained unchanged, and most interventions demonstrated similar patterns of SBP reduction relative to “no intervention” and “conventional health education”. The main difference observed was the enhanced SBP-lowering effect of “salt substitutes” when compared against both “no intervention” and “conventional health education”. Relative to “no intervention”, the estimated SBP reduction achieved with “salt substitutes” increased from −6.78 mmHg (95% CI −8.42 to −5.14) to −7.95 mmHg (95% CI −9.90 to −6.00). When compared with “conventional health education”, the SBP reduction increased from −5.35 mmHg (95% CI −7.89 to −2.81) to −6.45 mmHg (95% CI −9.06 to −3.84) (Supplementary Figure S2). Although point estimates increased, the confidence intervals overlapped substantially, indicating a modest strengthening of the estimated benefit of salt substitutes following outlier exclusion, while overall conclusions remain unchanged. In addition, the results of the quality assessment are provided in Supplementary Table S6. None of the 42 studies analysed were classified as low quality, meaning that all received five or more “yes” responses. These findings indicate that the inclusion of all studies in the network meta-analysis yielded reliable statistical outcomes.

The network meta-analysis results were further compared with the corresponding pairwise meta-analyses (Supplementary Table S10) to assess the consistency of effect estimates. Direct and indirect evidence showed consistent direction across key comparisons, indicating consistency within the network and supporting the robustness of the conclusions. For comparisons of “salt substitutes” with conventional health education or no intervention, pairwise meta-analyses yielded slightly greater SBP reductions—approximately 1 mmHg—than the corresponding estimates from the network meta-analysis. For the comparison of low-sodium diets combined with health education versus no intervention, the network meta-analysis yielded a slightly greater SBP reduction—approximately 1 mmHg—than the corresponding pairwise meta-analysis. Digital health education compared with conventional health education also showed higher SBP reductions in the network meta-analysis. Four comparisons appeared only in the network meta-analysis, including digital health education and self-monitoring plus health education versus no intervention, offering valuable additional information for comparing interventions. This approach shows that the network meta-analysis provided additional evidence absent from the pairwise meta-analysis results.

The heterogeneity (*I*^2^) observed in the subgroup pairwise meta-analyses ranged from 0% to 97%, indicating substantial variability among the included trials (Supplementary Table S8). As the commonly referenced cutoff values of 25%, 50%, and 75% for *I*^2^ indicate minor, moderate, and significant heterogeneity, the results suggested significant heterogeneity. The test for most subgroup differences was not statistically significant; however, the observed heterogeneity and the small number of studies per subgroup limited the interpretability of these estimates.

## 4. Discussion

In this systematic review and network meta-analysis of 42 studies (46 trials, *n* = 46,771) published over the past 25 years, we found among that seven identified interventions, “*salt substitutes*”*,* “*low-sodium diets*” with “*conventional health education*”, and “*self-monitoring devices*” with “*conventional health education*” were identified as the most effective approaches to reducing SBP in populations with elevated blood pressure, including those with hypertension. Our results suggest that self-regulated interventions—including self-monitoring devices and low-sodium diets combined with conventional health education—achieved systolic blood pressure reductions of a magnitude similar to those observed with salt substitutes. Salt substitutes, nevertheless, ranked highest in the network meta-analysis, reducing SBP by 6.78 mmHg (95% CI, –8.42 to –5.14) compared with no intervention and by 5.35 mmHg (95% CI, –7.89 to –2.81) compared with conventional health education. “*Digital health education*” showed a larger point estimate than “*conventional health education*” alone; however, the confidence interval was wide and crossed the null, indicating substantial uncertainty. In contrast, “*conventional health education*” alone demonstrated minimal effect on SBP reduction. Health education, whether it incorporates technology or not, may not significantly reduce SBP in populations with elevated blood pressure.

Despite the demonstrated effects and challenges of implementing salt substitutes, our network meta-analysis suggests that the combination of “*low-sodium diets*” and “*conventional health education*” may achieve SBP reductions of a similar magnitude to those achieved by salt-substitute use. Our findings showed a −6.58 mmHg reduction compared with no intervention, a slightly larger effect than the −5.2 mmHg reported by Siervo et al. in the general population [25], highlighting this approach as a potential alternative to salt-substitute strategies. Without “*conventional health education*”, “*low-sodium diets*” showed significantly lower reductions in SBP of about 1 to 1.5 mmHg. Compared with “*conventional health education*”, “*low-sodium diets*” combined with “*conventional health education*” reduced SBP by 5.15 mmHg, a similar magnitude to that achieved with the use of salt substitutes (−5.35 mmHg). Our findings indicate a viable alternative to salt substitutes, particularly in clinical settings where dietary management is already practiced and where concerns regarding the health effects of salt substitutes may restrict their adoption. Subgroup analyses indicated a statistically significant difference by trial duration; however, this finding should be interpreted with caution. Although trials lasting >3 to <6 months showed numerically larger systolic blood pressure reductions than those >6 months, the number of studies within each subgroup was small and heterogeneity was substantial. Therefore, these results should be considered exploratory and do not provide strong evidence of a true duration-dependent effect. For more sustainable outcomes, broader dietary reform efforts are necessary, such as reformulating food and beverages to reduce the sodium content, which can be implemented at individual, institutional, and national levels [89]. At the national level, successful implementation requires active engagement from private-sector food producers and the enforcement of regulatory standards [90]. As noted by Ding et al., most salt reduction policies have targeted chain restaurants, often still neglecting smaller food service operators [91]. At the individual level, sodium reduction can be supported by providing guidance to eliminate high-sodium items and adopt alternative low-sodium seasonings [92]. The lower-sodium DASH diet, for example, has shown significant effects in reducing blood pressure at sodium intake levels below 1500 mg/day and offers benefits for both hypertensive and pre-hypertensive populations [93].

Our findings also indicate that the combination of “*self-monitoring devices*” and “*conventional health education*” was associated with mean reductions of 6.81 mmHg (95% CI −13.41 to −0.22) and 5.38 mmHg (95% CI −11.64 to 0.88) when compared to “*no intervention*” and “*conventional health education*”, respectively, outperforming the reduction of 2.45 mmHg reported by Hisamatsu et al. in a broader population that included non-hypertensive individuals [26]. These findings underscore the potential of self-monitoring tools, including salt meters, to improve dietary awareness and facilitate behavioural change. By increasing users’ sensitivity to salt and providing tangible feedback, these devices can facilitate education on daily intake adherence to sodium-reduction goals. The salt meter has been shown to enhance taste sensitivity and promote dietary changes over time [76]. However, its utilization is limited, particularly in clinical settings and for hypertensive patients receiving care at home [94]. Nonetheless, our analysis included a limited number of trials with low heterogeneity, which restricts the generalizability of our findings. Additional research is needed to evaluate the long-term effectiveness of these tools across diverse hypertensive subgroups and healthcare systems.

In line with prior evidence, our network meta-analysis ranked “*salt substitutes*” as having the greatest SBP-reducing impact. Without the outliers and the PP trial, our sensitivity network meta-analysis also demonstrated greater systolic blood pressure (SBP) reductions via the use of “salt substitutes” compared with both “no intervention” and “conventional health education”, with an approximate additional reduction of 1 mmHg. These findings support previous research and guidelines that present salt substitution as both a crucial dietary guideline and a scalable, cost-effective public health strategy [14,95] that is particularly suitable for individuals with high sodium consumption and pharmaceutical treatment limitations. Nevertheless, our subgroup analysis demonstrated a statistically significant difference in SBP reductions across country income levels, with high-income countries showing a modest tendency toward larger effects. Implementation remains challenging in resource-limited settings, where equitable access to kidney screening and adequate iodization are limited [96], and in higher-income settings, where frequent eating out and concerns about the safety of salt substitutes hinder adoption.

By contrast, “*digital health education*”—delivered through smartphone applications, websites, or other web-based platforms—showed smaller effect estimates than salt substitutes and for self-regulated interventions combined with health education. Our network meta-analysis showed a reduction of 3.59 mmHg (95% CI −7.40 to 0.22) compared to “*no intervention*” and 2.16 mmHg compared to “*conventional health education*” (95% CI −5.37 to 1.04). In our network meta-analysis, the SBP reduction compared with ‘no intervention’ exceeded, by a modest margin, the −2.67 mmHg change previously reported by Yan et al. in their meta-analysis of the use of digital sodium-reduction tools across diverse populations [97]. However, the confidence intervals for both comparisons crossed the null, indicating substantial uncertainty and suggesting that the true effect may be modest or negligible. Conversely, “*conventional health education*”*,* consisting of counselling on nutrition and material provision, showed minimal effects on SBP reduction, with a reduction of 1.43 mmHg (95% CI −3.49 to 0.63) compared to “*no intervention*”. Extending a prior systematic review by Silva-Santos et al. [27] that qualitatively examined the effectiveness of health education in reducing sodium intake, our study quantified the magnitude of its effect on SBP. However, the confidence intervals for both comparisons crossed the null, indicating substantial uncertainty and suggesting that the true effect may be modest or negligible. Our subgroup analysis also suggested that trial duration and intervention setting may influence SBP reduction, with 3–6-month trials and clinical settings tending to yield larger effects. Nevertheless, when investigating the statistical subgroup difference between clinical and community settings for “conventional health education” versus “no intervention,” the community-based estimate indicated an increase in SBP, a finding driven mainly by a single study [87] with an upward SBP effect. The variability in trial results underscores the need for future interventions to incorporate clearer educational components, more standardised implementation protocols, and strengthened monitoring frameworks to minimise heterogeneity in community settings. Given a larger point estimate of systolic blood pressure (SBP) reduction compared with “*conventional health education*”, “*digital health education*” may constitute a more effective adjunct when integrated with self-regulated behavioural interventions. Nevertheless, the wide and overlapping confidence intervals reflect considerable uncertainty, highlighting the need for further investigation through additional trials. Factors such as age, digital literacy, and comorbidities may influence the effectiveness of digital health tools and should be considered in intervention design and deployment. However, the impact of combining digital health education with self-regulated strategies merits further investigation.

Our subgroup pairwise analyses suggested that the intervention effects were generally consistent across treated and untreated populations with elevated blood pressure. Most clinical factors (blood pressure stage, trial duration, and medication status) and public health factors (intervention setting, implementation period, and country income level) did not show statistically significant effect modification across most comparisons. However, given the limited number of studies within several subgroups and the substantial heterogeneity observed, these findings should be interpreted cautiously and may not provide definitive evidence of broad generalizability. Beyond sodium intake, broader behavioural factors, along with genetic, environmental, socio-economic, and psychosocial factors, contribute to the pathophysiology of elevated blood pressure and hypertension. While our review focused on dietary sodium-related interventions, other non-pharmaceutical behavioural approaches—such as increased physical activity, stress reduction, limited alcohol intake, weight management, and reduced exposure to air pollution—also play critical behavioural roles in preventing hypertension [98].

To our knowledge, this is the first network meta-analysis to comprehensively evaluate sodium-reduction interventions exclusively among populations with elevated blood pressure, including individuals with hypertension, enabling both direct and indirect comparisons across multiple intervention types. By restricting inclusion to randomized controlled trials, we ensured a high level of methodological rigor and internal validity. Furthermore, the inclusion of studies conducted over a 25-year period (2000–2025) provides historical depth and captures evolving intervention strategies across diverse settings.

However, several limitations should be acknowledged. First, the total number of included studies and studied populations was modest (42 studies with 46 trials, *n* = 46,771). Although we followed guidance to include a minimum of two studies for intervention in the analysis [42], some comparisons were based on only two studies with limited sample sizes, which reduced statistical power and the robustness of subgroup analyses. Consequently, these findings should be interpreted with caution. Second, although ITT analyses were prioritized, some outcome data were derived from PP analyses, which may introduce bias related to adherence or attrition. Sensitivity analyses revealed no meaningful differences between ITT and PP subgroups for most comparisons, except for salt substitutes versus no intervention. Notably, the single PP trial reported a substantially larger effect than the ITT trials (−19.71 vs. −6.36 mmHg), likely reflecting higher adherence and its extended 3-year duration. Although a sensitivity network meta-analysis excluding salt-substitute outliers (including one PP trial) yielded estimates of similar magnitude to the primary analysis, these findings highlight that adherence may play a critical role in maximizing intervention effectiveness. Third, the studies included in this review varied in context, including blood pressure stage, trial duration, medication status, intervention setting, implementation period, and country income level. Although most subgroup differences were not statistically significant, residual heterogeneity may have persisted, restricting the interpretability of the subgroup estimates. Fourth, most studies were conducted in high- and upper-middle-income countries, with limited representation of low-income countries. This restricts the generalizability of our findings to resource-limited contexts. Fifth, fifteen studies (34.88%) were conducted in China and Hong Kong. Of these, 11 of 14 studies (78.57%) examined salt substitutes compared to no intervention. Given the concentration of salt-substitute trials in Upper-Middle Income Countries, the evidence is largely driven by studies from East Asia. The generalizability of this subgroup analysis to regions with differing dietary patterns may be limited. However, in light of the limited adoption of salt substitutes in many high-income countries, including the United States [21], and given our objective to identify feasible alternative interventions, we regard these data as appropriate and relevant for our analysis. Sixth, heterogeneity (*I*^2^) within subgroups ranged widely (*I*^2^ = 0–95%), reflecting substantial variation across the included trials. Given the differing levels of heterogeneity, interpretation of individual subgroup effects is limited when substantial within-subgroup heterogeneity is present, aside from the formal test for subgroup differences. Seventh, to meet the minimum requirement of having at least two studies per comparison in a network meta-analysis and to preserve estimability across all treatment contrasts, we included three abstract-only studies that met the revised inclusion criteria. These abstracts provided all essential numerical data for effect-size calculation, including the mean SBP change, sample sizes, intervention comparisons, and key trial characteristics such as duration and setting. Nonetheless, the absence of full methodological reports limited the depth of the risk-of-bias assessment. Excluding two of these abstracts (one for the “self-monitoring devices” combined with “health education” vs. “conventional health education” comparison and one for the “salt substitutes” vs. “conventional health education” comparison) would have reduced the number to fewer than two contributing trials, preventing valid estimation and disrupting network connectivity. For the same reason, the remaining abstract-only study in the “low-sodium diet” combined with “health education” vs. “conventional health education” comparison was also retained to avoid selective exclusion that would create an unbalanced or inconsistent application of the inclusion criteria. Their inclusion maintains analytic validity, and we note that involving these nodes should be interpreted cautiously. Finally, our focus on community-level, bottom-up interventions means that policy-level, top-down approaches—such as regulatory bans, fiscal tools, or national food reformulation mandates—were outside the scope of this review. Nevertheless, we believe that highlighting community-based strategies offers valuable insight into scalable, population-level interventions for intensive SBP control among targeted populations with elevated blood pressure, including individuals with hypertension.

## Data Availability

The original data presented in the study are available in Table S3, which summarizes the included studies and trials.

## Supplementary Materials

The following supporting information can be downloaded at https://www.mdpi.com/article/10.3390/nu18030428/s1. Table S1: The details of the search strategy and results; Table S2: PICO Inclusion and exclusion criteria; Table S3: Summary of the included studies; Table S4: R code and description of network meta-analysis; Table S5: Characteristics of the included studies; Table S6: Quality assessment; Table S7: Summary of subgroup analysis; Table S8: Subgroup analysis; Table S8.1: Subgroup analysis 1—Type of Trials; Table S8.2: Subgroup analysis 2—Blood pressure stage; Table S8.3: Subgroup analysis 3—Trial duration; Table S8.4: Subgroup analysis 4—Medication status; Table S8.5: Subgroup analysis 5—Type of settings; Table S8.6: Subgroup analysis 6—Implementation period; Table S8.7: Subgroup analysis 4—Country income level; Table S9: Sensitivity analysis—outlier identification and analysis; Table S10: Pairwise meta-analysis of sodium-reduction interventions on mean SBP reduction in populations with elevated blood pressure; Table S11: PRISMA Checklist; Figure S1: Funnel plots for each pairwise meta-analysis; Figure S1.1: Funnel plot—“salt substitutes” and “no intervention”; Figure S1.2: Funnel plot—“low-sodium diets” and “no intervention”; Figure S1.3: Funnel plot—“low-sodium diets” with “conventional health education” and “conventional health education”; Figure S1.4: Funnel plot—“digital health education” and “conventional health education”; Figure S1.5: Funnel plot—“conventional health education” and “no intervention”; Figure S1.6: Funnel plot—“self-monitoring devices for urinary salt excretion” with “conventional health education on salt reduction” and “conventional health education on salt reduction”; Figure S1.7: Funnel plot—“salt substitutes” and “conventional health education on salt reduction”; Figure S2: Sensitivity network meta-analysis without outliers (Supplementary Table S9) and one per-protocol “salt substitutes vs. no intervention” trial. Ref. [99] was cited in Supplementary Materials.
