# Supplementary material for: Comparative Effectiveness of Behavioural Sodium-Reduction Interventions for Intensive Systolic Blood Pressure Control in Populations with Elevated Blood Pressure: A Systematic Review and Network Meta-Analysis
Source: Nutrients. 2026 Jan 28;18(3):428. doi: 10.3390/nu18030428 (PMC12899601; doi:10.3390/nu18030428)
Supplement: Supplementary file 1 [file nutrients-18-00428-s001.zip › nutrients-4014684-supplementary.pdf]

Supplementary Materials

A. Supplementary Tables

Supplementary Table S1: The details of the search strategy and results

Supplementary Table S2: PICO Inclusion and exclusion criteria

Supplementary Table S3: Summary of the included studies

Supplementary Table S4: R code and description of network meta-analysis

Supplementary Table S5: Characteristics of the included studies

Supplementary Table S6: Quality assessment

Supplementary Table S7: Summary of subgroup analysis

Supplementary Table S8: Subgroup analysis

- Supplementary Table S8.1: Subgroup analysis 1 - Type of Trials
- Supplementary Table S8.2: Subgroup analysis 2 - Blood pressure stage
- Supplementary Table S8.3: Subgroup analysis 3 - Trial duration
- Supplementary Table S8.4: Subgroup analysis 4 – Medication status
- Supplementary Table S8.5: Subgroup analysis 5 - Type of settings
- Supplementary Table S8.6: Subgroup analysis 6 – Implementation period
- Supplementary Table S8.7: Subgroup analysis 4 - Country income level

Supplementary Table S9: Sensitivity analysis – outlier identification and analysis

Supplementary Table S10: Pairwise meta-analysis of sodium-reduction interventions on mean SBP reduction in populations with elevated blood pressure

Supplementary Table S11: PRISMA Checklist

B. Supplementary Figures

Supplementary Figure S1: Funnel plots for each pairwise meta-analysis

Supplementary Figure S1.1: Funnel plot – "salt substitutes" and "no intervention"

Supplementary Figure S1.2: Funnel plot – "low-sodium diets" and "no intervention"

Supplementary Figure S1.3: Funnel plot – "low-sodium diets" with "conventional health education" and "conventional health education"

Supplementary Figure S1.4: Funnel plot – "digital health education" and "conventional health education"

Supplementary Figure S1.5: Funnel plot – "conventional health education" and "no intervention"

Supplementary Figure S1.6: Funnel plot – "self-monitoring devices for urinary salt excretion" with "conventional health education on salt reduction" and "conventional health education on salt reduction"

Supplementary Figure S1.7: Funnel plot – "salt substitutes" and "conventional health education on salt reduction"

Supplementary Figure S2: Sensitivity network meta-analysis without outliers (Supplementary Table S9) and one per-protocol "salt substitutes vs no intervention" trial

Supplementary Table S1 The details of the search strategy

("salt reduction" OR "sodium reduction" OR "salt intake", "sodium intake" OR "salt consumption" OR "sodium consumption") AND "hypertension" OR "high blood pressure" OR "systolic blood pressure" OR “diastolic blood pressure” OR "hypertensive disorder”) AND ("trial" OR "randomized controlled trial")

Supplementary Table S2 PICO Inclusion and exclusion criteria

Inclusion criteria	Inclusion criteria	Exclusion criteria
**Participants**	Adults aged 18 years or older with elevated blood pressure (SBP ≥120 mmHg, including those in the pre-hypertensive range).	Adults with systolic blood pressure (SBP) <120 mmHg and all participants aged below 18 years.
**Intervention**	Primary studies evaluating sodium-reduction interventions in real-world or experimental settings.	Systematic reviews and primary studies focused on population-level sodium-reduction initiatives or reformulation efforts targeting processed-food manufacturers.
**Comparators**	Primary studies where the control interventions were compared, including no intervention, usual care, or conventional health education	Primary studies where no comparison with control groups.

Inclusion criteria	Inclusion criteria	Exclusion criteria
**Outcomes**	Mean systolic blood pressure (SBP) reduction between intervention and control groups at the end of randomized controlled trials (RCTs) among eligible populations.	Other outcomes, such as reductions in daily dietary salt intake (g/day) or decreases in SBP without intervention–control comparison, as well as evaluations lacking quantitative data.
**Study design**	Primary studies with randomized control trial (RCT) including multi-arm trials.	Systematic reviews, primary studies with non-randomized controlled designs (non-RCTs), commentary or opinion articles, and qualitative studies lacking quantitative assessment were excluded.
**Study and publication periods**	Studies conducted and published between 2000 and February 2025 were eligible if full-texts were available or, in the case of abstracts, if sufficient information was provided; only studies written in English were included.	Studies conducted or published before 2000 or after February 2025, studies available only as abstracts with insufficient information for eligibility screening, and studies written in languages other than English were excluded.

Supplement Table S3 Summary of the included papers

Trial No.	Author	Year of Publication	Intervention		Mean Difference in SBP	Type of Trial	Number of Participants		Studied Country		Population						Intervention Explanation (Intervention Group)			Control Group	Studied Method	Studied Period	Study Period (Before or After May 2013)	Trial Period
			Intervention	Control			Intervention	Control	Country	Income Level	BP Level	Hypertension Stage	Other Diagnosed diseases	Age (Mean SD)/ F %	Setting	Medication Use	Intervention Group	Used of Technology?	Public Health Strategy					
1	Allaert	2013	Salt substitute	Conventional health education	-6.30	ITT	20	20	France	HIC	N/A	Hypertension (Diagnosis)	ND	N/A	Clinical-	No	NaC1 (standard marine salt) with Chitosan 3%	No	Dietary Intervention	No Intervention (Regular salt)	RCT (Double-blinded, Crossover)	N/A	Before	<=3 Months
2	Allaert	2017	Salt substitute	No Intervention	-11.50	ITT	22	19	France	HIC	BP = 130-140/ 80-89 mmHg	Elevated blood pressure (Diagnosis)	ND	51 (7)/ F 48.8%	Community	No	NaC1 (standard marine salt) with Chitosan 3%	No	Dietary Intervention	No Intervention (Regular salt)	RCT (Double-blinded, Monocenter)	N/A	After	<=3 Months
3	Appel	2003	Low-sodium diets with conventional health education	Conventional health education	-6.30	ITT	269	273	USA	HIC	BP = 120-159 X 80-95 mmHg	Hypertension (Diagnosis)	ND	Intervention: 50.2 (9.3)/ F 57.2% Vs. Control: 49.5 (8.8)/ F 63%	Clinical	No	DASH Diet with Health Education	No	Clinical Care Guideline /Health Promotion	Conventional Health Education	RCT (Single-blinded)	Jan 2000 - June 2001	Before	>3 to 6 Months
4	Azadbhakht	2005	Low-sodium diets	No Intervention	- -10.43	ITT	38	40	Iran	UMIC	BP >= 130/85 mmHg	Elevated blood pressure (Diagnosis)	Metabolic Syndrome	Intervention: 41.5 (12.3)/ F 71% Vs. Control: 41.3 (12.1)/ F 70%	Clinical	No	DASH Diet	No	Clinical Care Guideline	No intervention (Usual Controlled Diet)	RCT	N/A	Before	>3 to 6 Months
5	Barros	2015	Salt substitute	Conventional health education	-6.43*	ITT	19	16	Brazil	UMIC	BP >= 140x90 mmHg	Hypertension (Diagnosis)	ND	55.5 (7.4)/ 65.7%	Clinical	Yes	Light Salt	No	Dietary Intervention	No Intervention (Regular salt)	RCT (Single-blinded)	May 2021 - October 2021	After	<=3 Months
6	Blumentha	2010	Low-sodium diets	No Intervention	-7.80**	PP	46	48	USA	HIC	BP = 130-159/ 85-99 mmHg	Hypertension (Diagnosis)	ND	Intervention: 51.8 (10)/ F 63% Vs. Control: 51.8 (9)/ F = 69%	Clinical	No	DASH Diet	No	Clinical Care Guideline	No intervention (Usual Controlled Diet)	RCT (Assessor-blinded)	N/A (Enrolment - October 2003-July 2005 with 4-month treatment period)	Before	>3 to 6 Months
7	Hu	2018	Salt substitute	No Intervention	-4.20*	ITT	110	110	China	UMIC	BP >140/90 mmHg	Hypertension (Diagnosis)	ND	Intervention: 57.6 (10.1) / F 60% Vs. Control: 57.1 (10.9)/ F 66.4%	Clinical	ND	Salt substitute	No	Dietary Intervention	No Intervention (Regular salt)	RCT	Around Sep 2005	Before	>6 to 12 Months
8	Brito-Ashurst	2013	Low-sodium diets with conventional health education	Conventional health education	-8.00	PP	25	23	UK	HIC	BP = >=130/80 mmHg	Elevated blood pressure (Diagnosis)	Chronic Kidney Disease	Intervention: 55.7 (15.1) F 44% Vs. Control: 60.7 (12) F 39.13%	Clinical	ND	DASH Diet	No	Clinical Care Guideline/ Health Promotion	No Intervention (Reduced salt diet coached by dietarian)	RCT (Parallel-group)	June 2008 - July 2010	Before	>6 to 12 Months

Trial No.	Author	Year of Publication	Intervention		Mean Difference in SBP	Type of Trial	Number of Participants		Studied Country		Population						Intervention Explanation (Intervention Group)			Control Group	Studied Method	Studied Period	Study Period (Before or After May 2013)	Trial Period
			Intervention	Control			Intervention	Control	Country	Income Level	BP Level	Hypertension Stage	Other Diagnosed diseases	Age (Mean SD)/ F %	Setting	Medication Use	Intervention Group	Used of Technology?	Public Health Strategy					
9	Chen	2024	Low-sodium diets	No Intervention	-10.10	ITT	27	26	China	UMIC	SBP >130-159 mmHg	Elevated blood pressure (Determined)	ND	Intervention: Age 30-59 48.1%/ Age >=60 51.9%/ F 59.3% Vs. Control: Age 30-59 53.8%/ Age >= 60 46.2%/ F 34.6%	Community	Yes	Chinese heart-healthy diet (CHH)	No	Dietary Intervention	No intervention (Usual Controlled Diet)	RCT	March 2019 - Jan 2021	After	<=3 Months
10	CSSS	2007	Salt substitute	No Intervention	-3.70	ITT	292	293	China	UMIC	SBP >= 160 mmHg	Hypertension (Diagnosis)	ND	Intervention: 59 (10) F 48% Vs. Control: 61 (9.7) F 58%	Community	ND	Salt substitute (reduced-sodium and high-potassium)	No	Dietary Intervention	No Intervention (Regular salt)	RCT (Double-Blinded)	May 2004 - August 2005	Before	> 12 Months
11	D'Elia	2025	Conventional health education	No Intervention	-0.98	ITT	109	121	Italy	HIC	ND	Hypertension (Diagnosis)	ND	Intervention: 57.5 (10.6) F 46% Vs. Control: 57.5 (10.6) F 43%	Clinical	Yes	Dietary Educational Intervention by physician	No	Health Education	No Intervention (No Education)	RCT (Multi-Center)	ND	After	<= 3 Months
12	Ding	2025	Salt substitute	No Intervention	-2.05	ITT	7703	7546	China	UMIC	ND	Elevated blood pressure (Diagnosis)	Stroke	Intervention: 64.0 (8.80 F 46.1% Vs. Control: 64.2 (8.8) F 45.7%	Community	ND	Salt substitute	No	Dietary Intervention	No Intervention (Regular salt)	RCT (Cluster)	ND	After	> 12 Months
13	Dorsch	2020	Digital health education	Conventional health education	-6.80*	ITT	24	26	USA	HIC	SBP < 180/110 mmHg	Hypertension (Diagnosis)	ND	Intervention: 56.6 (10) F 58% Vs. Control: 58.2 (11) F 61%	Clinical	Yes	Mobile Application for high-sodium food assessment	Yes	Health Education	No Intervention (No Application)	RCT (Single-Center)	June2017 - March 2019	After	<= 3 Months
14	Eyles	2017	Digital health education	Conventional health education	1.00	ITT	33	33	New Zealand	HIC	ND	Elevated blood pressure (Determined)	CVD	64 (7)/ F 17%	Community	ND	Mobile Application to support lower-salt food purchase	Yes	Health Education	Conventional Health Education from usual care	RCT (Two-arm parallel)	June 2014 - July 2015	After	<= 3 Months
15	Eyles	2023	Digital health education	Conventional health education	-0.66*	ITT	84	84	New Zealand	HIC	>= 140/85 mmHg	Hypertension (Determined)	ND	Intervention: 54 (13) F 45% Vs. Control: 55 (13)/ F 43%	Community	No	Salt Switch Smart Phone Application	Yes	Health Education	Conventional Health Education (Generic heart-healthy eating information)	RCT (Two-arm parallel)	June 2019 - Feb 2020	After	<= 3 Months

Trial No.	Author	Year of Publication	Intervention		Mean Difference in SBP	Type of Trial	Number of Participants		Studied Country		Population						Intervention Explanation (Intervention Group)			Control Group	Studied Method	Studied Period	Study Period (Before or After May 2013)	Trial Period
			Intervention	Control			Intervention	Control	Country	Income Level	BP Level	Hypertension Stage	Other Diagnosed diseases	Age (Mean SD)/ F %	Setting	Medication Use	Intervention Group	Used of Technology?	Public Health Strategy					
16	Ferrante	2011	Low-sodium diets	No Intervention	-1.70	ITT	29	29	Argentina	UMIC	SBP >= 120-159 mmHg	Elevated blood pressure (Determined)	ND	Intervention: 41.5 (12.4)/ F 62.9% Vs. Control: 37.1 (9.1)/ F 78.5%	Community	Yes	Reduced Salt Bread	No	Dietary Intervention	No Intervention (Normal Salt Bread)	RCT (2 x 2 Crossover)	2006-2007	Before	<= 3 Months
17	Filippou	2023	Low-sodium diets with conventional health education	Conventional health education	-10.10	ITT	60	60	USA	HIC	ND	Hypertension (Diagnosis)	ND	Intervention: 50.9 (12.7)/ F 50% Vs. Control: 53.2 (11)/ F 50%	Clinical	No	DASH Diet with Health Education	No	Clinical Care Guideline /Health Promotion	Conventional Health Education (General advice on dietary principles)	RCT (Single-Center, Single-blinded)	ND	After	<= 3 Months
18	Golshahi - A	2015	Conventional health education	No Intervention	-7.96	ITT	45	15***	Iran	UMIC	BP =140-158/90-99 mmHg	Hypertension (Diagnosis)	ND	Intervention: 56.7 (6.7)/ F 78% Vs. Control:57.7 (8.5)/ F 80%	Clinical	No	Health Education on Self-Care by Cardiologist	No	Health Education	No Intervention (Usual Health Education on Care of Hypertension)	RCT (Prospective)	July - December 2013	After	>3 to 6 Months
19	Golshahi - B	2015	Conventional health education	No Intervention	-1.98	ITT	45	15***	Iran	UMIC	BP =140-158/90-99 mmHg	Hypertension (Diagnosis)	ND	Intervention: 57.44 (9.2)/ F 75.6% Vs. Control:57.7 (8.5)/ F 80%	Clinical	No	Self-Education through four pamphlets	No	Health Education	No Intervention (Usual Health Education on Care of Hypertension)	RCT (Prospective)	July - December 2014	After	>3 to 6 Months
20	Golshahi - C	2015	Conventional health education	No Intervention	-0.91	ITT	45	15***	Iran	UMIC	BP =140-158/90-99 mmHg	Hypertension (Diagnosis)	ND	Intervention: 56.76 (8.9) / F 80.00% Vs. Control:57.7 (8.5)/ F 80%	Clinical	No	Self-Education through SMS	Yes	Health Education	No Intervention (Usual Health Education on Care of Hypertension)	RCT (Prospective)	July - December 2015	After	>3 to 6 Months
21	Gupta	2023	Low-sodium diets	No Intervention	-8.00**	ITT	95	118	USA	HIC	BP =90-160/50-100 mmHg	Elevated blood pressure (Determined)	ND	Intervention: 61 (Median 58-65) / F 66% Vs. Control:61 (Median 56-64)/ F 64%	Community	No	Low-Sodium Diet	No	Dietary Intervention	No Intervention (High-Sodium Diet)	RCT	Enrolment: April 2021 and Feb 2023/ Trial - ND	After	<= 3 Months
22	Hu	2009	Salt substitute	No Intervention	-6.30	PP	93	94	China	UMIC	Hypertension (Diagnosis) (1) Coronary Cerebral or peripheral vascular disease OR (2) Diabetes and aged 55 or older OR (3) SBP >= 160 mmHg (Hypertension Grade 2 or above)			Intervention: 59 (10.0) / F 54% Vs. Control: 59 (9.1)/ F65%	Community	ND	Salt substitute	No	Dietary Intervention	No Intervention (Regular Salt)	RCT (Double-Blinded)	May 2004 - Aug 2005	Before	<= 3 Months

Trial No.	Author	Year of Publication	Intervention		Mean Difference in SBP	Type of Trial	Number of Participants		Studied Country		Population						Intervention Explanation (Intervention Group)			Control Group	Studied Method	Studied Period	Study Period (Before or After May 2013)	Trial Period
			Intervention	Control			Intervention	Control	Country	Income Level	BP Level	Hypertension Stage	Other Diagnosed diseases	Age (Mean SD)/ F %	Setting	Medication Use	Intervention Group	Used of Technology?	Public Health Strategy					
23	Humalda	2020	Digital health education	Conventional health education	-4.70	PP	41	39	The Netherlands	HIC	BP> 135/85 mmHg	Hypertension (Diagnosis)	Chronic Kidney Disease	Intervention: 55.1 (11.5) / F 14% Vs. Control: 58.2 (13.2)/ F 18%	Clinical	Yes	Routine Care with Web-based Self Management	Yes	Health Education	No Intervention (Routine Care)	RCT	June 2014 - March 2015	After	>6 to 12 Months
24	AM Irwan - A	2016	Conventional health education	No Intervention	-3.30	PP	13	8.5***	Indonesia	UMIC	BP > 120/80 mmHg	Hypertension (Diagnosis)	ND	Intervention: 67.9 (6.9) / F 46.2% Vs. Control:66.1 (5.7)/ F 70.6%	Community	ND	Health Education (Salt Reduction Training)	No	Health Education	No Intervention (Routine Care)	RCT	Oct 2014 - Jan 2015	After	>3 to 6 Months
25	AM Irwan - B	2016	Conventional health education	No Intervention	-1.40	PP	15	8.5***	Indonesia	UMIC	BP > 120/80 mmHg	Hypertension (Diagnosis)	ND	Intervention: 65.8 (4.9) / F 66.7% Vs. Control:66.1 (5.7)/ F 70.6%	Community	ND	Health Education (Salt Reduction and Efficacy Maintenance)	No	Health Education	No Intervention (Routine Care)	RCT	Oct 2014 - Jan 2016	After	>3 to 6 Months
26	Lai	2024	Salt substitute	No Intervention	-7.14	ITT	807	805	China	UMIC	Mean SBP 137.5 (21.3)/ Mean DBP 80.5 (11.6)	Elevated blood pressure (Determined)	ND	71.0 (9.5)/ F 23.7%	Care (48 Elderly Care Facilities)	ND	Salt Substitute	No	Dietary Intervention	No Intervention (Regular Salt)	RCT	Sep 2017 - Oct 2022	After	> 12 Months
27	Liu	2022	Digital health education	Conventional health education	-3.99	ITT	111	115	China	UMIC	BP >= 140 x 90 mmHg	Hypertension (Determined)	ND	Intervention: 48.58 (9.54) / F 43.7% Vs. Control: 50.64 (8.72)/ F 53%	Clinical	ND	mHealth App	Yes	Health Education	No Intervention (Routine Care)	RCT (Parallel and Prospective)	April 2017-Jan 2019	After	> 12 Months
28	Naseem	2016	Low-sodium diets with conventional health education	Conventional health education	-2.00	PP	782	710	Pakistan	LMIC	N/A	Hypertension (Diagnosis)	ND	N/A	Clinical	Yes	Low-sodium diet with Health Education	No	Dietary Intervention/ Health Education	Health Education	RCT	Feb 2014- March 2015	After	> 12 Months
29	Neal	2021	Salt substitute	No Intervention	-3.34	ITT	10504	10491	China	UMIC	SBP >160 mmHg (> 140 mmHg with Med)	Hypertension (Diagnosis)	Stroke	Mean Age 65.4/ F 49.5%	Community	No	Salt substitute	No	Dietary Intervention	No Intervention (Regular Salt)	RCT (Open-label, Cluster)	Enrolment: April 2014 - Jan 2015/ Trial: Completion in March 2021	After	> 12 Months

Trial No.	Author	Year of Publication	Intervention		Mean Difference in SBP	Type of Trial	Number of Participants		Studied Country		Population						Intervention Explanation (Intervention Group)			Control Group	Studied Method	Studied Period	Study Period (Before or After May 2013)	Trial Period
			Intervention	Control			Intervention	Control	Country	Income Level	BP Level	Hypertension Stage	Other Diagnosed diseases	Age (Mean SD)/ F %	Setting	Medication Use	Intervention Group	Used of Technology?	Public Health Strategy					
30	Malloy-Mcfall	2010	Low-sodium diets	No Intervention	-9.00	ITT	10	10	USA	HIC	BP = 120-160/ 80-95 mmHg	Hypertension (Determined)	ND	Intervention: 38.5 (10.5)/ F 50% Vs. Control: 38.1 (11.1)/ F 30%	Community (School)	ND	DASH Diet	No	Dietary Intervention	No Intervention (Regular Diet)	RCT	ND	Before	<= 3 Months
31	Morikawa	2011	Self-monitoring device with Conventional health education	Conventional health education	-3.20*	ITT	22	19	Japan	HIC	N/A	Hypertension (Determined)	N/A	N/A	N/A	ND	N/A	N/A	N/A	N/A	N/A	N/A	N/A	<= 3 Months
32	Mu	2022	Low-sodium diets	No Intervention	-0.28	PP	30	29	China	UMIC	ND	Hypertension (Diagnosis)	Type 2 Diabetes	Intervention: 70 (4.24) / F63.3% Vs. Control: 68.03 (3.82)/ F 51.7%	Clinical	Yes	Low-Sodium DASH Diet	No	Clinical Care Guideline	No Intervention (Regular Diet)	RCT (Single-blinded)	July - December 2020	After	<= 3 Months
33	Petersen	2013	Conventional health education	No Intervention	-2.00	PP	31	35	Australia	HIC	ND	High-risk hypertension	Type 2 Diabetes		Clinical	ND	Dietary Education on low-sodium processed foood	No	Health Education	No Intervention (No Education)	RCT	July - Dec 2011	Before	<= 3 Months
34	Rahimdel	2019	Conventional health education	No Intervention	2.09	PP	60	67	Iran	UMIC	BP >120-139/ 80-89 mmHg	High-risk hypertension	ND	Intervention: 42.43 (4.9) / F 57.1% Vs. Control: 42.61 (5.5) / F 61.4%	Community	No	Health Education (Salt Reduction Training based on theory of planned behavior)	No	Health Education	No Intervention (No Education)	RCT	Feb - Dec 2017	After	>6 to 12 Months
35	SP Riches	2021	Digital health education	Conventional health education	0.10	PP	29	16	UK	HIC	SBP > 130 mmHG (on Medication) or > 140 mmHg (Without Medication)	Hypertension (Diagnosis)	ND	Intervention: 64 (12) / F 65% Vs. Control: 67 (7) / F 63%	Clinical-Setting	Yes	Mobiel Health Salt Intervention with Health Education	Yes	Health Education	Conventional Health Education (Usual Care with Salt Booklet)	RCT (Parallel, 2-arm)	2017	After	<= 3 Months
36	Sarkkinen	2011	Salt substitute	No Intervention	-12.00	ITT	22	23	Finland	HIC	BP =130-139/85-99 mmHg	Elevated blood pressure (Determined)	ND	Intervention: 57 (12) / F 59% Vs. Control: 54 (11)/ F39.13%	Community-Setting	No	Smart Salt	No	Dietary Intervention	No Intervention (Regular Salt)	RCT (Double-blinded)	ND	Before	<= 3 Months

Trial No.	Author	Year of Publication	Intervention		Mean Difference in SBP	Type of Trial	Number of Participants		Studied Country		Population						Intervention Explanation (Intervention Group)			Control Group	Studied Method	Studied Period	Study Period (Before or After May 2013)	Trial Period
			Intervention	Control			Intervention	Control	Country	Income Level	BP Level	Hypertension Stage	Other Diagnosed diseases	Age (Mean SD)/ F %	Setting	Medication Use	Intervention Group	Used of Technology?	Public Health Strategy					
37	Sun	2023	Salt substitute	No Intervention	-19.71	ITT	133	126	China	UMIC	BP >= 140 x 90 mmHg	Hypertension (Diagnosis)	ND	49.51/ F 50.2%	Clinical-Setting	ND	Low-Sodium Salt	No	Dietary Intervention	No Intervention (Regular Salt)	RCT (Double-Blinded)	April 2006 to April 2009	Before	> 12 Months
38	Sonuch	2024	Low-sodium diets with conventional health education	Conventional health education	-4.00	PP	111	108	Thailand	UMIC	BP >= 130/80 mmHg	Hypertension (Diagnosis)	ND	Intervention: 60 (7.5) / F81.1% Vs. Control:60.2 (7.5))/ F 72.2%	Clinical-Setting	No	Dietary Reormation with Health Education (Salt-monitoring devices provided)	Yes	Dietary Intervention/ Health Education	Conventional Health Education	RCT	Jan - June 2022	After	>3 to 6 Months
39	Wiriyatanakorn	2021	Self-monitoring device with Conventional health education	Conventional health education	-6.20	ITT	45	45	Thailand	UMIC	BP >= 140-180/90-110 mmHg	Hypertension (Diagnosis)	ND	Intervention: 63.2 (11.9) / F 58% Vs. Control:62.5 (10)/ F 51%	Clinical-Setting	Yes	Self-Monitoring Devices with Health Education	Yes	Health Education	Conventional Health Education	RCT (Single-Center, Open-Labelled)	June 2017 - Jan 2020	After	<= 3 Months
40	MCS Wong	2015	Low-sodium diets with conventional health education	Conventional health education	-0.24	PP	243	242	China (Hong Kong)	UMIC	BP = 140-159/ 90-99 mmHg	Hypertension (Diagnosis)	ND	Intervention: 55.4 (5.6) / F 53.6% Vs. Control:54.9 (5.2)/ F 48.4%	Clinical-Setting	No	DASH with Health Education	No	Dietary Intervention/ Health Education	Conventional Health Education	RCT (Parallel-group)	Jan - June 2013	Between	>6 to 12 Months
41	Yang - A	2018	Salt substitute	No Intervention	-10.18	ITT	24	27	China	UMIC	SBP > 140 and DBP < 90 mmHg or ISH diagnosed	Hypertension (Diagnosis)	ND	Intervention: 67.8 (5.34) / F 58.3% Vs. Control:65.9 (6.17)/ F 59.2%	Community-Setting	Yes	Low-Sodium Salt	No	Dietary Intervention	No Intervention (Regular Salt)	RCT (Single-blinded)	ND	After	>6 to 12 Months
42	Yang- B	2018	Salt substitute	No Intervention	-5.10	ITT	38	37	China	UMIC	DBP > 90 mmHg or NISH diagnosed	Hypertension (Diagnosis)	ND	Intervention: 67.3 (5.62) / F 55.3% Vs. Control:65.4 (6.75)/ F 56.8%	Community-Setting	Yes	Low-Sodium Salt	No	Dietary Intervention	No Intervention (Regular Salt)	RCT (Single-blinded)	ND	After	>6 to 12 Months
43	Yuan	2023	Salt substitute	No Intervention	-7.fi10	ITT	807	805	China	UMIC	ND	Elevated blood pressure (Determined)	ND	Intervention: 71.6 (9.8) / F 26.1% Vs. Control:70.5 (9.2)/ F 21.1%	Care-Setting (48 elderly care facilities)	ND	Salt Substitute	No	Dietary Intervention	No Intervention (Regular Salt)	RCT (Cluster 2x2 design)	ND	After	> 12 Months

Trial No.	Author	Year of Publication	Intervention		Mean Difference in SBP	Type of Trial	Number of Participants		Studied Country		Population						Intervention Explanation (Intervention Group)			Control Group	Studied Method	Studied Period	Study Period (Before or After May 2013)	Trial Period
			Intervention	Control			Intervention	Control	Country	Income Level	BP Level	Hypertension Stage	Other Diagnosed diseases	Age (Mean SD)/ F %	Setting	Medication Use	Intervention Group	Used of Technology?	Public Health Strategy					
44	Zhao	2014	Salt substitute	No Intervention	-7.60	ITT	141	141	China (Tibet)	LMIC	SBP >= 140 mmHg	Hypertension (Determined)	ND	Intervention: 62.8 (11.1) / F 60.3% Vs. Control:63.5 (11.3)/ F 57.4%	Community-Setting	ND	Salt Substitute	No	Dietary Intervention	No Intervention (Regular Salt)	RCT	Dec 2008 - Feb 2009	Before	>6 to 12 Months
45	Zhou	2009	Salt substitute	No Intervention	-11.80	ITT	62	64	China	UMIC	SBP >= 140 mmHg	Hypertension (Determined)	ND	Intervention: 65.7 (6.3) / F 56.5% Vs. Control: 65.7(6.3)/ F 57.8%	Community-Setting	No	Low-Sodium Salt (Compound Ion Salt)	No	Dietary Intervention	No Intervention (Regular Salt)	RCT (Single-Blinded)	Sep 2003 - May 2005	Before	<= 3 Months
46	Zhou	2016	Salt substitute	No Intervention	-11.29	ITT	224	238	China	UMIC	ND	Hypertension (Determined)	ND	Intervention: 45.63 (13.72) / F 49.55% Vs. Control: 47.05 (13.46)/ F 49.16%	Community-Setting	ND	Low-Sodium Salt	No	Dietary Intervention	No Intervention (Regular Salt)	RCT (Double-Blinded)	ND	After	> 12 Months

Remark

1. PP* - ITT was reported in the study but with incompleted data (such as losses to follow-up, missing studied outcomes etc.
2. Mean SBP differences were reported with the office SBP approach. The home SBP approach measured mean SBP differences with *, while the ambulatory SBP approach measured those with **.
3. Participants from multi-arm trials were handled according to Cochrane guidance for pairwise meta-analysis to avoid double counting with ***.

Supplementary Table S4: R code and description of network meta-analysis

4.1) R code description:

**Item**	**Description**
Software	R version 4.3.2
Packages	Netmeta 2.9-0, dplyr 1.1.4
Effect measure	Mean difference
Model	Graph-theoretical network meta-analysis incorporating pairwise comparisons
Ranking method	Netrank (P-scores; larger P-scores indicate higher ranking, with smaller effect estimates indicating better outcomes)

4.2) R code for network meta-analysis:

4.2.1) Software environment

```
#	Load required packages
library(readxl)
library(dplyr)
library(tidyr)
library(netmeta)
```

4.2.2) Data import

```
raw_data <- read_excel(
  "R104052025_Dataset.xlsx",
```

4.2.3) Data preparation

```
data <- raw_data %>%
  rename(
    study_id     = `Trial No.`,
    author       = Author,
    year         = `Year of Publication`,
    intervention = Intervention...5,
    TE           = `Mean Difference`,
    seTE         = SE,
    n1           = Control,
```

```
    n2          = `Intervention...10`
  ) %>%
  separate(
    intervention,
    into = c("treat1", "treat2"),
    sep = "-",
    extra = "merge",
    fill = "right",
    remove = FALSE
  ) %>%
  mutate(
    treat1  = trimws(as.character(treat1)),
    treat2  = trimws(as.character(treat2)),
    TE      = as.numeric(TE),
    seTE    = as.numeric(seTE),
    n1      = as.numeric(n1),
    n2      = as.numeric(n2),
    studlab = as.character(study_id)
  ) %>%
  filter(
    !is.na(treat1), !is.na(treat2),
    treat1 != "", treat2 != "",
    !is.na(TE), !is.na(seTE),
    seTE > 0,
    !is.na(studlab)
  )
```

4.2.4) Network meta-analysis

```
netmeta_obj <- netmeta(
  TE = TE,
  seTE = seTE,
  treat1 = treat1,
  treat2 = treat2,
  studlab = studlab,
  n1 = n1,
  n2 = n2,
  data = data,
  sm = "MD"
)
```

4.2.5) Visualisation from network ranking and forest plots

```
netgraph(
```

```r
  netmeta_obj,
  thickness = "number.of.studies",
  multiarm = TRUE,
  seq = netmeta_obj$trts,
  col.points = "blue",
  col = "black",
  points = TRUE,
  cex.points = 3,
  cex = 0.9,
  plastic = FALSE,
  number.of.studies = TRUE,
  lwd = 2
)

forest(netmeta_obj)
if ("HE" %in% netmeta_obj$trts) forest(netmeta_obj, ref = "HE")
if ("NOT" %in% netmeta_obj$trts) forest(netmeta_obj, ref = "NOT")

rankings <- netrank(netmeta_obj, small.values = "good")
plot(rankings, main = "Ranking of Treatments")
print(rankings)
```

4.2.6) Funnel plot analysis and outlier detection

```r
#     Prepare funnel plots.
ord <- c("DHE", "LD", "LD+HE", "HE", "NOT", "SMD+HE", "SS")
ord <- ord[ord %in% netmeta_obj$trts]
par(cex = 0.7)
funnel_data <- funnel(
  netmeta_obj,
  order = ord,
  contour = TRUE,
  contour.levels = c(0.1, 0.05, 0.01),
  plot = FALSE
)
par(cex = 1)

#Assign x and y from the correct columns.
x <- funnel_data$TE.adj  # centred effect (x-axis)
y <- funnel_data$seTE    # standard error (y-axis)

#Compute 95% confidence triangle bounds.
upper_bound <-  1.96 * y
lower_bound <- -1.96 * y
```

```
#Identify outliers and create the table.
idx_out <- which(x < lower_bound | x > upper_bound)

outliers <- data.frame(
  studlab = funnel_data$studlab[idx_out],
  treat1  = funnel_data$treat1[idx_out],
  treat2  = funnel_data$treat2[idx_out],
  TE_adj  = x[idx_out],
  seTE    = y[idx_out],
  lower   = lower_bound[idx_out],
  upper   = upper_bound[idx_out]
)

print(outliers)
```

Supplementary Table S5 Characteristics of the included studies

a) **Studied countries**

High-income countries (HICs)		17 Studies
	USA	6 studies
	UK	2 studies
	New Zealand	2 studies
	France	2 studies
	Australia	1 study
	Finland	1 study
	Italy	1 study
	Japan	1 study
	The Netherland	1 study
Upper-middle income countries (UMICs)		23 Studies
	China + Hong Kong	15 studies
	Iran	3 studies
	Thailand	2 studies
	Argentina	1 study
	Brazil	1 study
	Indonesia	1 study
Low- and middle-income countries (LMICs)		2 Studies
	Pakistan	1 study
	Tibet (China)	1 study

b) Studied periods

Before May 2013	16 studies
After May 2013	25 studies
Between May 2013	1 study

c) Comparative trials

Salt substitutes Vs no interventions	14 studies (16 trials)
Low-sodium diets Vs no interventions	7 studies (7 trials)
Low-sodium diets Vs Conventional health education	6 studies (6 trials)
Conventional health education Vs no interventions	5 studies (8 trials)
Digital health education Vs Conventional health education	6 studies (6 trials)
Salt substitutes Vs Conventional health education	2 studies (2 trials)

d) Quality ratings

High quality	20 studies
Moderate quality	19 studies
Low quality	0 study
Not Applicable	3 studies (availability of the full-text version)

Supplementary Table S6 Quality assessment

	Author	Year of Publication	Q1 True randomization	Q2 Conceal allocation to treatment groups	Q3 Similarity of treatment groups at baseline	Q4 Participants blinded to treatment assignment	Q5 Those delivering treatment blinded to treatment assignment	Q6 Outcome assessors blinded to treatment assignment	Q7 Treatment groups treated identically other than the intervention of interes	Q8 Differences between groups and their follow up adequately described and analyzed	Q9 Participants analyzed in the groups to which they were randomized	Q10 Outcomes measured in the same way for treatment groups	Q11 Outcomes measured in a reliable way	Q12 Appropriate statistical analysis used	Q13 Appropriatness of trial design	Quality Grading
1	Allaert	2013	*	*	*	*	*	*	*	*	*	*	*	*	*	N/A
2	Allaert	2017	X	X	O	O	X	X	O	O	O	O	UC	O	O	Medium
3	Appel	2003	*	*	*	*	*	*	*	*	*	*	*	*	*	N/A
4	Azadbhakht	2005	O	O	O	UC	X	O	O	O	O	O	O	O	O	High
5	Barros	2015	X	X	O	O	X	X	O	UC	X	O	UC	O	O	Medium
6	Blumentha	2010	O	O	O	O	O	O	UC	UC	O	O	O	O	O	High

	Author	Year of Publication	Q1 True randomization	Q2 Conceal allocation to treatment groups	Q3 Similarity of treatment groups at baseline	Q4 Participants blinded to treatment assignment	Q5 Those delivering treatment blinded to treatment assignment	Q6 Outcome assessors blinded to treatment assignment	Q7 Treatment groups treated identically other than the intervention of interes	Q8 Differences between groups and their follow up adequately described and analyzed	Q9 Participants analyzed in the groups to which they were randomized	Q10 Outcomes measured in the same way for treatment groups	Q11 Outcomes measured in a reliable way	Q12 Appropriate statistical analysis used	Q13 Appropriatness of trial design	Quality Grading
7	Brito-Ashurst	2013	O	X	O	X	X	O	UC	UC	X	O	O	O	O	Medium
8	Chen	2024	O	UC	O	O	UC	UC	O	O	O	O	O	O	O	High
9	CSSS	2007	O	O	O	O	O	O	O	O	O	O	O	O	O	High
10	D'Elia	2025	O	O	O	O	UC	UC	O	O	O	O	O	O	O	High
11	Ding	2025	UC	UC	O	X	X	O	UC	O	O	O	O	O	O	Medium
12	Dorsch	2020	O	UC	O	UC	UC	UC	UC	X	X	O	X	O	O	Medium
13	Eyles	2017	O	O	O	X	UC	X	UC	O	O	O	UC	O	O	High

	Author	Year of Publication	Q1 True randomization	Q2 Conceal allocation to treatment groups	Q3 Similarity of treatment groups at baseline	Q4 Participants blinded to treatment assignment	Q5 Those delivering treatment blinded to treatment assignment	Q6 Outcome assessors blinded to treatment assignment	Q7 Treatment groups treated identically other than the intervention of interes	Q8 Differences between groups and their follow up adequately described and analyzed	Q9 Participants analyzed in the groups to which they were randomized	Q10 Outcomes measured in the same way for treatment groups	Q11 Outcomes measured in a reliable way	Q12 Appropriate statistical analysis used	Q13 Appropriatness of trial design	Quality Grading
14	Eyles	2023	O	X	O	X	X	X	X	O	O	O	O	O	O	Medium
15	Ferrante	2011	O	O	O	O	O	O	O	O	O	O	O	O	O	High
16	Filippou	2023	O	O	O	O	O	UC	UC	O	O	O	O	O	O	High
17	Golshahi	2015	X	UC	O	X	X	X	UC	O	O	O	UC	O	O	Medium
18	Gupta	2023	UC	UC	UC	UC	UC	UC	O	X	O	O	O	O	O	Medium
19	Hu	2009	O	O	O	O	O	O	O	O	O	O	O	O	O	High
20	Hu	2018	O	O	O	O	O	O	O	O	O	O	UC	O	O	High

	Author	Year of Publication	Q1<br>True randomization	Q2<br>Conceal allocation to treatment groups	Q3<br>Similarity of treatment groups at baseline	Q4<br>Participants blinded to treatment assignment	Q5<br>Those delivering treatment blinded to treatment assignment	Q6<br>Outcome assessors blinded to treatment assignment	Q7<br>Treatment groups treated identically other than the intervention of interes	Q8<br>Differences between groups and their follow up adequately described and analyzed	Q9<br>Participants analyzed in the groups to which they were randomized	Q10<br>Outcomes measured in the same way for treatment groups	Q11<br>Outcomes measured in a reliable way	Q12<br>Appropriate statistical analysis used	Q13<br>Appropriatness of trial design	Quality Grading
21	Humalda	2020	O	O	O	O	O	UC	UC	O	X	O	UC	O	O	High
22	Irwan	2016	O	O	O	O	O	O	UC	O	X	O	UC	O	O	High
23	Lai	2024	O	O	UC	UC	UC	O	O	O	O	O	O	O	O	High
24	Liu	2022	O	O	O	O	O	UC	UC	O	O	O	UC	O	O	High
25	Naseem	2016	O	O	O	UC	X	X	UC	O	X	O	UC	O	O	Medium
26	Neal	2021	O	O	O	X	X	O	O	O	O	O	O	O	O	High
27	Malloy-Mcfall	2010	O	UC	O	UC	UC	UC	O	UC	UC	O	UC	O	O	Medium

	Author	Year of Publication	Q1 True randomization	Q2 Conceal allocation to treatment groups	Q3 Similarity of treatment groups at baseline	Q4 Participants blinded to treatment assignment	Q5 Those delivering treatment blinded to treatment assignment	Q6 Outcome assessors blinded to treatment assignment	Q7 Treatment groups treated identically other than the intervention of interes	Q8 Differences between groups and their follow up adequately described and analyzed	Q9 Participants analyzed in the groups to which they were randomized	Q10 Outcomes measured in the same way for treatment groups	Q11 Outcomes measured in a reliable way	Q12 Appropriate statistical analysis used	Q13 Appropriatness of trial design	Quality Grading
28	Morikawa	2011	*	*	*	*	*	*	*	*	*	*	*	*	*	N/A
29	Mu	2022	O	UC	O	O	X	X	O	O	X	O	O	O	O	High
30	Petersen	2013	X	X	O	UC	X	X	O	O	X	O	O	O	O	Medium
31	Rahimdel	2019	O	UC	O	UC	X	X	X	O	X	O	O	O	O	Medium
32	SP Riches	2021	O	O	UC	X	X	X	O	O	X	O	UC	O	O	Medium
33	Sarkkinen	2011	UC	UC	O	O	O	O	O	UC	X	O	O	O	O	High
34	Sun	2023	O	UC	X	O	O	O	UC	X	X	O	O	O	O	Medium

	Author	Year of Publication	Q1 True randomization	Q2 Conceal allocation to treatment groups	Q3 Similarity of treatment groups at baseline	Q4 Participants blinded to treatment assignment	Q5 Those delivering treatment blinded to treatment assignment	Q6 Outcome assessors blinded to treatment assignment	Q7 Treatment groups treated identically other than the intervention of interes	Q8 Differences between groups and their follow up adequately described and analyzed	Q9 Participants analyzed in the groups to which they were randomized	Q10 Outcomes measured in the same way for treatment groups	Q11 Outcomes measured in a reliable way	Q12 Appropriate statistical analysis used	Q13 Appropriatness of trial design	Quality Grading
35	Sonuch	2024	O	X	O	UC	UC	UC	UC	O	X	O	O	O	O	
36	Wiriyatanakorn	2021	O	UC	O	UC	UC	UC	UC	UC	X	O	UC	O	O	Medium
37	Wong	2015	O	X	O	X	X	X	UC	O	O	O	UC	O	O	Medium
38	Yang	2018	UC	UC	O	UC	UC	UC	O	UC	UC	O	O	O	O	Medium
39	Yuan	2023	O	O	O	X	X	O	O	O	O	O	O	O	O	High
40	Zhao	2014	O	UC	O	O	X	UC	O	O	O	O	O	O	O	High
41	Zhou	2009	O	UC	O	O	X	X	O	O	O	O	O	O	O	High

	Author	Year of Publication	Q1 True randomization	Q2 Conceal allocation to treatment groups	Q3 Similarity of treatment groups at baseline	Q4 Participants blinded to treatment assignment	Q5 Those delivering treatment blinded to treatment assignment	Q6 Outcome assessors blinded to treatment assignment	Q7 Treatment groups treated identically other than the intervention of interes	Q8 Differences between groups and their follow up adequately described and analyzed	Q9 Participants analyzed in the groups to which they were randomized	Q10 Outcomes measured in the same way for treatment groups	Q11 Outcomes measured in a reliable way	Q12 Appropriate statistical analysis used	Q13 Appropriatness of trial design	Quality Grading
42	Zhou	2016	O	O	X	O	UC	O	O	O	UC	O	UC	O	O	High

Supplementary Table S7 Summary of subgroup difference analysis (P subgroup > 0.05)

Intervention Comparison		Total		No. of studies	No. of trials	Clinical Factors				Public Health Factors		
Intervention	Control	Intervention	Control			Stage of blood	Trial duration	Medication Usage	Type of studied trials	Intervention setting	Period of study	Country income level
Salt substitutes	No intervention	20,982	20,819	14	15	X	X	X	X	X	X	O
Salt substitutes	Conventional health education	39	36	2	2	ND	ND	ND	ND	ND	ND	ND
Low-sodium diets	No intervention	275	300	7	7	X	X	X	X	X	X	X
Low-sodium diets with conventional health education	Conventional health education	1,490	1,416	6	6	X	O	X	X	X	X	X
Self-monitoring devices with Conventional health education	Conventional health education	67	64	2	2	ND	ND	ND	ND	ND	ND	ND
Digital health education	Conventional health education	322	313	6	6	X	X	NR	X	X	X	X
Conventional health education	No intervention	363	384	5	8	X	O	NR	X	O	X	X

Interpretation: O – Statistical subgroup difference

X – No statistical subgroup difference

ND – Not defined due to limited number of trials

NR – Not relevant

Supplementary Table S8 Subgroup analysis

Supplementary Table S8.1 Subgroup analysis - Type of trials

Comparison	Type of Trials	Number of Trials	Mean Difference (SBP)	P Value	I2
Low-sodium diets Vs No Intervention	Intention-to-treat	5	-7.09	0.0006	78%
	Per-protocol	2	-3.74	0.32	92%
	Intention-to-treat Vs Per-protocol	Subgroup difference $\chi^2 = 0.61$, P = 0.43, $I^2 = 0\%$ (Consistent intervention effects across ITT and PP trial types)			
Low-sodium diets with conventional health education Vs No intervention	Intention-to-treat	2	-8.07	<0.0001	70%
	Per-protocol	4	-3.57	0.002	77%
	Intention-to-treat Vs Per-protocol	Subgroup difference $\chi^2 = 3.05$, P = 0.08, $I^2 = 67.2\%$ (Consistent intervention effects across ITT and PP trial types)			

Comparison	Type of Trials	Number of Trials	Mean Difference (SBP)	P Value	I2
Digital health education Vs Conventional health education	Intention-to-treat	4	-1.45	0.23	0%
	Per-protocol	2	-2.72	0.25	1%
	Intention-to-treat Vs Per-protocol	Subgroup difference $\chi^2 = 0.23$, $P = 0.63$, $I^2 = 0\%$ (Consistent intervention effects across ITT and PP trial types)			
Salt substitutes Vs No Intervention	Intention-to-treat	14	-6.36	<0.00001	79%
	Per-protocol	1	-19.71	<0.0001	N/A
	Intention-to-treat Vs Per-protocol	Subgroup difference $\chi^2 = 0.00$, $P = 0.001$*, $I^2 = 90.7\%$ (Statistical subgroup difference between ITT and PP trial types)			

Comparison	Type of Trials	Number of Trials	Mean Difference (SBP)	P Value	I2
Conventional health education Vs No Intervention	Intention-to-treat **	4	-2.84	0.27	73%
	Per-protocol **	4	-0.31	0.82	67%
	Intention-to-treat Vs Per-protocol	Subgroup difference $\chi^2 = 1.29$, $P = 0.26$, $I^2 = 22.7\%$ (Consistent intervention effects across ITT and PP trial types)			

*p-value of the subgroup difference below 0.05 indicating a statistically significant subgroup effect.

** Group training and individual counseling for health education. *** Material provision for health education

Supplementary Table S8.2 Subgroup analysis 2 – Blood pressure stage (Office SBP)

Comparison	Blood Pressure Stage	Number of Trials	Mean Difference (SBP)	P Value	I2
Low-sodium diets Vs No Intervention	Hypertension (Formal diagnosis)	2	-2.73	0.49	56%
	Elevated blood pressure (Formal diagnosis)	1	-10.43	0.0004	N/A
	Elevated blood pressure (Determined)	2	-5.42	0.19	88%
	Blood pressure stage comparison	Subgroup difference $\chi^2 = 2.66$, $P = 0.26$, $I^2 = 24.8\%$ (Consistent intervention effects across blood pressure stage)			

Comparison	Blood Pressure Stage	Number of Trials	Mean Difference (SBP)	P Value	I2
Low-sodium diets with conventional health education Vs No intervention	Hypertension (Formal diagnosis)	4	-3.19	0.03	69%
	Elevated blood pressure (Formal diagnosis)	2	-5.93	0.14	96%
	Blood pressure stage comparison	Subgroup difference $\chi^2 = 0.41$, $P = 0.52$, $I^2 = 0\%$ (Consistent intervention effects across blood pressure stage)			

Comparison	Blood Pressure Stage	Number of Trials	Mean Difference (SBP)	P Value	I2
Digital health education Vs Conventional health education	Hypertension (Formal diagnosis)	3	-3.3	0.13	0%
	Hypertension (Determined)	2	-1.49	0.30	13%
	Blood pressure stage comparison	Subgroup difference $\chi^2 = 0.87$, $P = 0.65$, $I^2 = 0\%$ (Consistent intervention effects across blood pressure stage)			

Comparison	Blood Pressure Stage	Number of Trials	Mean Difference (SBP)	P Value	I2
Salt substitutes Vs No Intervention	Hypertension (Formal diagnosis)	6	-7.25	0.03	89%
	Hypertension (Determined)	3	-10.00	0.02	0%
	Elevated blood pressure (Formal diagnosis)	3	-5.93	0.41	85%
	Elevated blood pressure (Determined)	2	-7.51	0.02	0%
	Blood pressure stage comparison	Subgroup difference $\chi^2 = 2.73$, $P = 0.43$, $I^2 = 0\%$ (Consistent intervention effects across blood pressure stage)			

Comparison	Blood Pressure Stage	Number of Trials	Mean Difference (SBP)	P Value	I2
Conventional health education Vs No Intervention	Hypertension (Formal diagnosis)	6	-1.58	0.003	21%
	High-risk hypertension (Determined)	2	-0.06	0.97	88%
	Blood pressure stage comparison	Subgroup difference $\chi^2 = 0.52$, P = 0.47, $I^2 = 0\%$ (Consistent intervention effects across blood pressure stage)			

* P-value of the subgroup difference below 0.05 indicating a statistically significant subgroup effect.

Supplementary Table S8.3 Subgroup analysis 3 – Trial duration (Office SBP)

Comparison	Trial Duration	Number of Trials	Mean Difference (SBP)	P Value	I2
Low-sodium diets Vs No Intervention	<= 3 Months	4	-3.99	0.10	97%
	> 3 Months to 6 Months	1	-10.43	0.0004	N/A
	Trial duration comparison	Subgroup difference $\chi^2 = 2.80$, P = 0.09, $I^2 = 64.2\%$ (Consistent intervention effects across trial durations)			
Low-sodium diets with conventional health education Vs No intervention	<= 3 Months	4	-3.19	0.03	69%
	> 3 Months to 6 Months	2	-5.51	<0.00001	5%
	> 6 Months to 12 Months	2	-4.09	0.29	93%
	> 12 Months	1	-2.00	0.29	93%
	Trial duration comparison	Subgroup difference $\chi^2 = 10.69$, P = 0.01*, $I^2 = 71.9\%$ (Statistically significant effects across trial durations)			

Comparison	Trial Duration	Number of Trials	Mean Difference (SBP)	P Value	I2
Digital health education Vs Conventional health education	<= 3 Months	2	0.44	0.88	0%
	> 3 Months to 6 Months	1	-4.70	0.12	N/A
	> 12 Months	1	-3.99	0.15	1N/A
	Trial duration comparison	Subgroup difference $\chi^2 = 1.83$, $P = 0.40$, $I^2 = 0\%$ (Consistent intervention effects across trial duration)			

Comparison	Trial Duration	Number of Trials	Mean Difference (SBP)	P Value	I2
Salt substitutes Vs No Intervention	<= 3 Months	4	-9.90	0.006	0%
	> 3 Months to 6 Months	3	-7.63	0.03	0%
	> 12 Months	7	-6.83	0.02	95%
	Trial duration comparison	Subgroup difference $\chi^2 = 1.97$, P = 0.37, $I^2 = 0\%$ (Consistent intervention effects across trial duration)			

Comparison	Trial Duration	Number of Trials	Mean Difference (SBP)	P Value	I2
Conventional health education Vs No Intervention	<= 3 Months	2	-1.78	0.15	0%
	> 3 Months to 6 Months	5	-2.53	0.11	74%
	> 6 Months to 12 Months	1	2.09	0.08	N/A
	Trial duration comparison	Subgroup difference χ^2 = 10.11, P = 0.006*, I^2 = 69%<br>(Statistically significant effects across trial durations)			

* P-value of the subgroup difference below 0.05 indicating a statistically significant subgroup effect.

Supplementary Table S8.4 Subgroup analysis 4 – Medication status (Office SBP)

Comparison	Medication Status	Number of Trials	Mean Difference (SBP)	P Value	I2
Low-sodium diets Vs No Intervention	Medication (Stable)	3	-3.36	0.21	98%
	No medication	1	-10.43	0.0004	N/A
	Not defined	1	-9.00	0.12	N/A
	Medication status comparison	Subgroup difference $\chi^2 = 3.27$, P = 0.20, $I^2 = 38.8\%$ (Consistent intervention effects across medical status)			
Low-sodium diets with conventional health education Vs No intervention	Medication	1	-2.00	0.32	N/A
	No medication	4	-5.55	0.01	87%
	Not defined	1	-6.30	< 0.00001	N/A
	Medication status comparison	Subgroup difference $\chi^2 = 3.25$, P = 0.20, $I^2 = 38.4\%$ (Consistent intervention effects across medical status)			

Comparison	Medication Status	Number of Trials	Mean Difference (SBP)	P Value	I2
Salt substitutes Vs No Intervention	Medication	2	-7.67	0.20	0%
	No medication	4	-8.69	0.03	74%
	Not defined	8	-7.25	0.004	95%
	Medication status comparison	Subgroup difference $\chi^2 = 0.25$, P = 0.88, $I^2 = 88\%$ (Consistent intervention effects across medical status)			

* P-value of the subgroup difference below 0.05 indicating a statistically significant subgroup effect.

Supplementary Table S8.5 Subgroup analysis 5 - Type of settings

Comparison	Type of Settings	Number of Trials	Mean Difference (SBP)	P Value	I2
"Low-sodium diets" and "no intervention"	Clinical	3	-5.66	0.07	90%
	Community	4	-6.35	0.006	80%
	Type of setting comparison	Subgroup difference $\chi^2 = 0.03$, $P = 0.86$, $I^2 = 0\%$ (Consistent intervention effects across intervention settings)			

Comparison	Type of Settings	Number of Trials	Mean Difference (SBP)	P Value	I2
"Low-sodium diets" with "conventional health education" and "conventional health education on salt reduction"	Clinical	6	-5.14	0.0008	82%
	Community	N/A	N/A	N/A	N/A
	Type of setting comparison	N/A			
"Salt substitutes" and "no intervention"	Clinical	2	-11.51	0.38	93%
	Community	11	-6.74	<0.01	84%
	Care Home	2	-7.12	<0.01	0%
	Type of setting comparison	Subgroup difference $\chi^2 = 0.43$, $P = 0.81$, $I^2 = 0\%$ (Consistent intervention effects across intervention settings)			

Comparison	Type of Settings	Number of Trials	Mean Difference (SBP)	P Value	I2
“Salt substitutes” and “conventional health education on salt reduction”	Clinical	2	-11.51	0.38	93%
	Community	11	-6.74	0.0002	85%
	Care home	2	-7.12	0.002	0%
	Type of setting comparison	Subgroup difference $\chi^2 = 0.43$, P = 0.81, $I^2 = 0\%$ (Consistent intervention effects across intervention settings)			

Comparison	Type of Settings	Number of Trials	Mean Difference (SBP)	P Value	I2
"Digital health education on sodium reduction" and "conventional health education on salt reduction"	Clinical	4	-3.56	0.04	0%
	Community	2	-0.52	0.71	0%
	Type of setting comparison	Subgroup difference $\chi^2 = 1.93$, $P = 0.17$, $I^2 = 48.1\%$ (Consistent intervention effects across intervention settings)			
"Conventional health education on salt reduction" and "no intervention"	Clinical	5	-1.63	0.04	9%
	Community	3	1.90	0.11	0%
	Type of setting comparison	Subgroup difference $\chi^2 = 16.27$, $P < 0.0001$*, $I^2 = 93.9\%$ (Statistically subgroup difference across intervention settings)			

*P-value of the subgroup difference below 0.05 indicating a statistically significant subgroup effect.

Supplementary Table S8.6 Subgroup analysis 6 – Implementation period

Comparison	Implementation Period	Number of Trials	Mean Difference (SBP)	P Value	I2
"Low-sodium diets" and "no intervention"	Before May 2013	4	-6.34	0.008	77%
	After May 2013	3	-5.68	0.07	92%
	Implementation period comparison	Subgroup difference Chi2 = 0.03, P = 0.87, I^2 = 0% (Consistent intervention effects between two periods)			
"Low-sodium diets" with "conventional health education" and "conventional health education on salt reduction"	Before May 2013	3	-4.82	0.04	88%
	After May 2013	3	-5.47	0.03	82%
	Implementation period comparison	Subgroup difference Chi2 = 0.04, P = 0.85, I^2 = 0% (Consistent intervention effects across two periods)			

Comparison	Implementation Period	Number of Trials	Mean Difference (SBP)	P Value	I2
“Digital health education on sodium reduction” and “conventional health education on salt reduction”	Before May 2013	N/A	N/A	N/A	N/A
	After May 2013	6	-1.71	0.11	0%
	Implementation period comparison	N/A			
“Salt substitutes” and “no intervention”	Before May 2013	7	-8.45	0.006	81%
	After May 2013	8	-6.35	0.001	86%
	Implementation period comparison	Subgroup difference Chi2 = 0.77, P = 0.38, I^2 = 0% (Consistent intervention effects across two periods)			

Comparison	Implementation Period	Number of Trials	Mean Difference (SBP)	P Value	I2
"Conventional health education on salt reduction" and "no intervention"	Before May 2013	1	-2.00	0.007	N/A
	After May 2013	7	-1.71	0.29	77%
	Implementation period comparison	Subgroup difference Chi2 = 0.03, P = 0.86, I^2 = 0% (Consistent intervention effects across two periods)			

*p-value of the subgroup difference below 0.05 indicating a statistically significant subgroup effect.

Supplementary Table S8.7 Subgroup analysis 7 - Country income level

Comparison	Country Income Level	Number of Trials	Mean Difference (SBP)	P Value	I2
"Low-sodium diets" and "no intervention"	High-income countries	3	-7.98	<0.00001	0%
	Upper-middle income countries	4	-4.95	0.06	98%
	Country Income level comparison	Subgroup difference Chi2 = 1.04, P = 0.31, I^2 = 3.7% (Consistent intervention effects across different income levels			

Comparison	Country Income Level	Number of Trials	Mean Difference (SBP)	P Value	I2
"Low-sodium diets" with "conventional health education" and "conventional health education on salt reduction"	High-income countries	4	-6.10	0.004	88%
	Upper-middle income countries	1	-4.00	0.03	N/A
	Low-income countries	1	-2.00	0.32	N/A
	Country Income level comparison	Subgroup difference Chi2 = 1.96, df = 2, P = 0.37, $I^2 = 0\%$ (Consistent intervention effects across different income levels			

Comparison	Country Income Level	Number of Trials	Mean Difference (SBP)	P Value	I2
"Digital health education on sodium reduction" and "conventional health education on salt reduction"	High-income countries	5	-1.31	0.26	0%
	Upper-middle income countries	1	-3.99	0.15	N/A
	Country Income level comparison	Subgroup difference Chi2 = 0.80, P = 0.37, I^2 = 0% (Consistent intervention effects across different income levels			

Comparison	Country Income Level	Number of Trials	Mean Difference (SBP)	P Value	I2
"Salt substitutes" and "no intervention"	High-income countries	2	-11.51	0.02	0%
	Upper-middle income countries (China)	12	-6.74	0.0003	89%
	Low-income countries	1	-7.19	0.002	N/A
	Country Income level comparison	Subgroup difference Chi2 = 14.22, P = 0.0008*, I^2 = 85.9% (Statistically subgroup difference across different income levels)			

Comparison	Country Income Level	Number of Trials	Mean Difference (SBP)	P Value	I2
"Conventional health education on salt reduction" and "no intervention"	High-income countries	2	-1.78	0.15	0%
	Upper-middle income countries	6	-2.16	0.31	82%
	Country Income level comparison	Subgroup difference Chi2 = 0.04, P = 0.84, I^2 = 0% (Consistent intervention effects across two income levels)			

*p-value of the subgroup difference below 0.05 indicating a statistically significant subgroup effect.

Supplementary Table S9 Sensitivity analysis – outlier identification and analysis

Outlier Identification

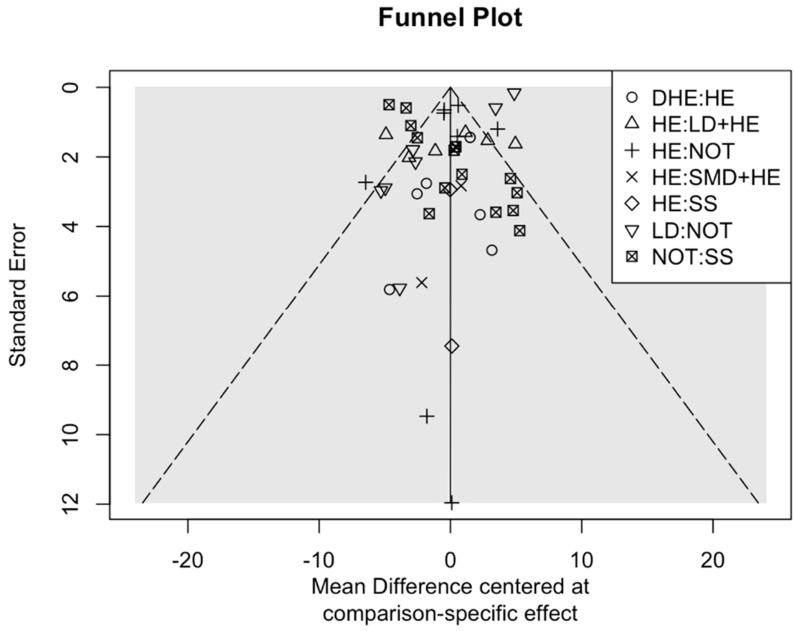

> outliers

	studlab	TE	TE.direct	TE.adj	seTE	pch	col
10	Ferrante_2011	-1.70	-5.138019	3.438019	0.590	2	black
13	Mu_2022	-0.28	-5.138019	4.858019	0.160	2	black
16	Filippou_2023	-10.10	-5.153373	-4.946627	1.630	3	black
19	MCS Wong_2015	-0.24	-5.153373	4.913373	1.360	3	black
21	Golshahi - A_2015	-7.96	-1.504341	-6.455659	2.730	4	black
27	Rahimdel_2019	2.09	-1.504341	3.594341	1.200	4	black
34	CSSS_2007	3.70	6.721003	-3.021003	1.100	7	black
35	Ding_2025	2.05	6.721003	-4.671003	0.500	7	black
38	Neal_2021	3.34	6.721003	-3.381003	0.594	7	black
40	Sun_2023	19.71	6.721003	12.988997	3.970	7	black

Outlier analysis

Comparison		With and without outliers	Subgroup Differences between ITT and PP (P-value)	Interpretation
"Low-sodium diets" and "no intervention"	LD - NOT	With outliers	0.43	Similar
		Without outliers	0.64	Similar
"Low-sodium diets" with "conventional health education" and "conventional health education"	LD + HE - HE	With outliers	0.08	Trend toward difference
		Without outliers	0.51	Similar
"Digital health education" and "conventional health education"	DHE - HE	With outliers	0.63	Similar
		Without outliers	0.63	Similar

Comparison		With and without outliers	Subgroup Differences between ITT and PP (P-value)	Interpretation
“Salt substitutes” and “no intervention”	SS - NOT	With outliers	0.001*	Statistical subgroup effect
		Without outliers	N/D**	N/D**
“Conventional health education” and “no intervention”	HE - NOT	With outliers	0.26	Similar
		Without outliers	0.59	Similar

*p-value of the subgroup difference below 0.05 indicating a statistically significant subgroup effect.

** Not applicable; all trials were ITT after outlier removal.

Supplementary Table S10: Pairwise meta-analysis of sodium-reduction interventions on mean SBP reduction in populations with elevated blood pressure

**Intervention comparison**		**Total**		**No. of studies**	**No. of trials**	**Pooled mean SBP difference (95% CI)**	**P-value**	**Heterogeneity I^2**
**Intervention**	**Control**	**Intervention**	**Control**					
Salt substitutes	No intervention	20,982	20,819	14	15	-7.19	<0.00001	86%
Salt substitutes	Conventional health education	39	36	2	2	-6.32	0.02	0%
Low-sodium diets	No intervention	275	300	6	6	-5.94	0.0007	95%
Low-sodium diets with conventional health education	Conventional health education	1,490	1,416	6	6	-5.14	0.0008	82%
Self-monitoring devices with Conventional health education	Conventional health education	67	64	2	2	-5.59	0.03	0%
Digital health education	Conventional health education	322	313	6	6	-1.71	0.11*	0%
Conventional health education	No intervention	363	284	5	8	-1.36	0.15*	69%

* The overall effect was tested using a Z-test; p <>0.05 indicates that the pooled effect is not statistically significant.

Supplementary Table S11 PRISMA Checklist

Section and Topic	Item #	Checklist item	Location where item is reported
**TITLE**			
Title	1	Identify the report as a systematic review.	1
**ABSTRACT**			
Abstract	2	See the PRISMA 2020 for Abstracts checklist.	3-4
**INTRODUCTION**			
Rationale	3	Describe the rationale for the review in the context of existing knowledge.	5-8
Objectives	4	Provide an explicit statement of the objective(s) or question(s) the review addresses.	8
**METHODS**			
Eligibility criteria	5	Specify the inclusion and exclusion criteria for the review and how studies were grouped for the syntheses.	9-10
Information sources	6	Specify all databases, registers, websites, organisations, reference lists and other sources searched or consulted to identify studies. Specify the date when each source was last searched or consulted.	9
Search strategy	7	Present the full search strategies for all databases, registers and websites, including any filters and limits used.	9
Selection process	8	Specify the methods used to decide whether a study met the inclusion criteria of the review, including how many reviewers screened each record and each report retrieved, whether they worked independently, and if applicable, details of automation tools used in the process.	10-11
Data collection process	9	Specify the methods used to collect data from reports, including how many reviewers collected data from each report, whether they worked independently, any processes for obtaining or confirming data from study investigators, and if applicable, details of automation tools used in the process.	10-11
Data items	10a	List and define all outcomes for which data were sought. Specify whether all results that were compatible with each outcome domain in each study were sought (e.g. for all measures, time points, analyses), and if not, the methods used to decide which results to collect.	10-11
	10b	List and define all other variables for which data were sought (e.g. participant and intervention characteristics, funding sources). Describe any assumptions made about any missing or unclear information.	10-11
Study risk of bias assessment	11	Specify the methods used to assess risk of bias in the included studies, including details of the tool(s) used, how many reviewers assessed each study and whether they worked independently, and if applicable, details of automation tools used in the process.	12
Effect measures	12	Specify for each outcome the effect measure(s) (e.g. risk ratio, mean difference) used in the synthesis or presentation of results.	10

Section and Topic	Item #	Checklist item	Location where item is reported
Synthesis methods	13a	Describe the processes used to decide which studies were eligible for each synthesis (e.g. tabulating the study intervention characteristics and comparing against the planned groups for each synthesis (item #5)).	12-15
	13b	Describe any methods required to prepare the data for presentation or synthesis, such as handling of missing summary statistics, or data conversions.	12-15
	13c	Describe any methods used to tabulate or visually display results of individual studies and syntheses.	12-15
	13d	Describe any methods used to synthesize results and provide a rationale for the choice(s). If meta-analysis was performed, describe the model(s), method(s) to identify the presence and extent of statistical heterogeneity, and software package(s) used.	12-15
	13e	Describe any methods used to explore possible causes of heterogeneity among study results (e.g. subgroup analysis, meta-regression).	14
	13f	Describe any sensitivity analyses conducted to assess robustness of the synthesized results.	13
Reporting bias assessment	14	Describe any methods used to assess risk of bias due to missing results in a synthesis (arising from reporting biases).	13
Certainty assessment	15	Describe any methods used to assess certainty (or confidence) in the body of evidence for an outcome.	13-15
**RESULTS**			
Study selection	16a	Describe the results of the search and selection process, from the number of records identified in the search to the number of studies included in the review, ideally using a flow diagram.	16
	16b	Cite studies that might appear to meet the inclusion criteria, but which were excluded, and explain why they were excluded.	16
Study characteristics	17	Cite each included study and present its characteristics.	17-23
Risk of bias in studies	18	Present assessments of risk of bias for each included study.	27-29
Results of individual studies	19	For all outcomes, present, for each study: (a) summary statistics for each group (where appropriate) and (b) an effect estimate and its precision (e.g. confidence/credible interval), ideally using structured tables or plots.	22-24
Results of syntheses	20a	For each synthesis, briefly summarise the characteristics and risk of bias among contributing studies.	24-27
	20b	Present results of all statistical syntheses conducted. If meta-analysis was done, present for each the summary estimate and its precision (e.g. confidence/credible interval) and measures of statistical heterogeneity. If comparing groups, describe the direction of the effect.	24-27
	20c	Present results of all investigations of possible causes of heterogeneity among study results.	27-29
	20d	Present results of all sensitivity analyses conducted to assess the robustness of the synthesized results.	27-29
Reporting biases	21	Present assessments of risk of bias due to missing results (arising from reporting biases) for each synthesis assessed.	N/A
Certainty of evidence	22	Present assessments of certainty (or confidence) in the body of evidence for each outcome assessed.	27-29

Section and Topic	Item #	Checklist item	Location where item is reported
**DISCUSSION**			
Discussion	23a	Provide a general interpretation of the results in the context of other evidence.	30-34
	23b	Discuss any limitations of the evidence included in the review.	35-37
	23c	Discuss any limitations of the review processes used.	35-37
	23d	Discuss implications of the results for practice, policy, and future research.	30-35
**OTHER INFORMATION**			
Registration and protocol	24a	Provide registration information for the review, including register name and registration number, or state that the review was not registered.	3 and 38
	24b	Indicate where the review protocol can be accessed, or state that a protocol was not prepared.	38
	24c	Describe and explain any amendments to information provided at registration or in the protocol.	N/A
Support	25	Describe sources of financial or non-financial support for the review, and the role of the funders or sponsors in the review.	38
Competing interests	26	Declare any competing interests of review authors.	38
Availability of data, code and other materials	27	Report which of the following are publicly available and where they can be found: template data collection forms; data extracted from included studies; data used for all analyses; analytic code; any other materials used in the review.	Supplementary Table S3

From: Page MJ, McKenzie JE, Bossuyt PM, Boutron I, Hoffmann TC, Mulrow CD, et al. The PRISMA 2020 statement: an updated guideline for reporting systematic reviews. BMJ 2021;372:n71. doi: 10.1136/bmj.n71. This work is licensed under CC BY 4.0. To view a copy of this license, visit https://creativecommons.org/licenses/by/4.0/

Supplementary Figure S1 Funnel plots for each pairwise meta-analysis

Supplementary Figure S1.1 Funnel plot – "salt substitutes" and "no intervention"

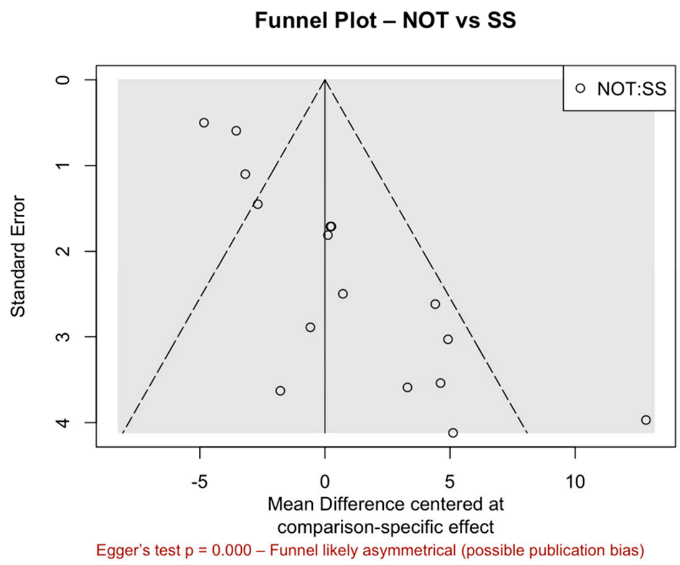

Supplementary Figure S1.2 Funnel plot – "low-sodium diets" and "no intervention"

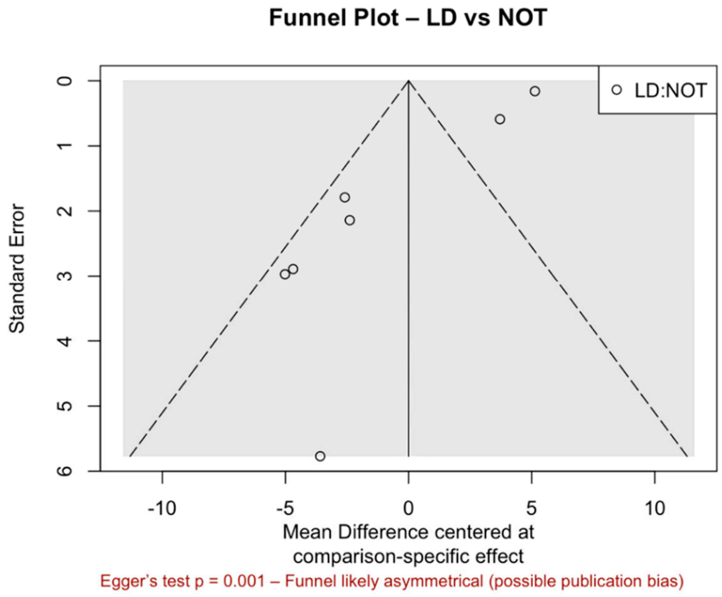

Supplementary Figure S1.3 Funnel plot – "low-sodium diets" with "conventional health education" and "conventional health education"

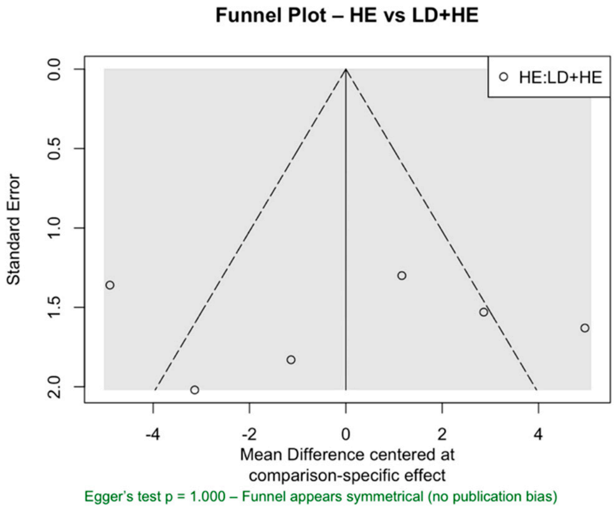

Supplementary Figure S1.4 Funnel plot – "digital health education" and "conventional health education"

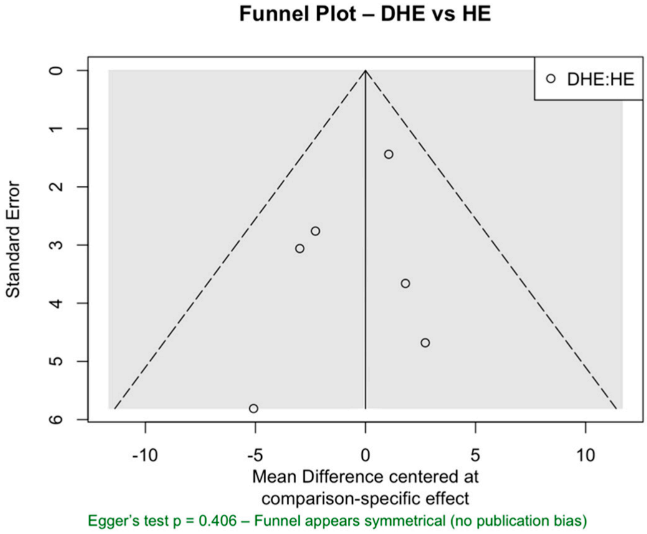

Supplementary Figure S1.5 Funnel plot – "conventional health education" and "no intervention"

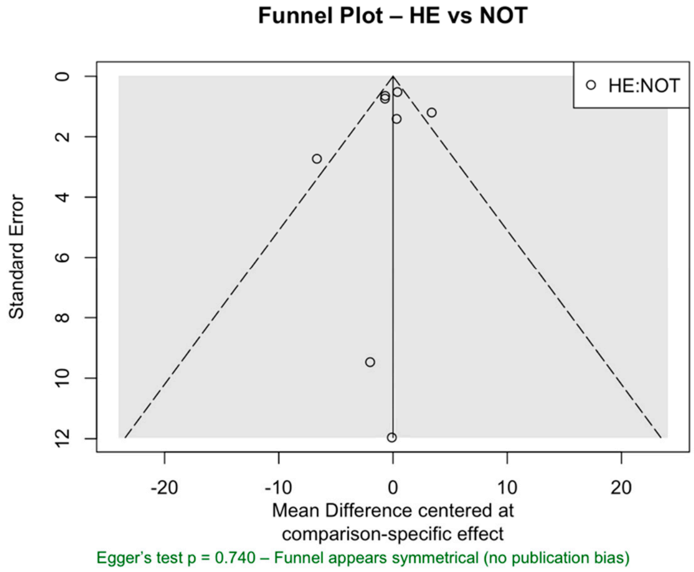

Supplementary Figure S1.6 Funnel plot – "self-monitoring devices for urinary salt excretion" with "conventional health education on salt reduction" and "conventional health education on salt reduction"

N/A as the included studies are less than three studies.

Supplementary Figure S1.7 Funnel plot – "salt substitutes" and "conventional health education on salt reduction"

N/A as the included studies are less than three studies.

Supplementary Figure S2: Sensitivity network meta-analysis without all outliers (Supplementary Table S9) and one per-protocol "salt substitutes vs no intervention" trial

a) Network ranking plot

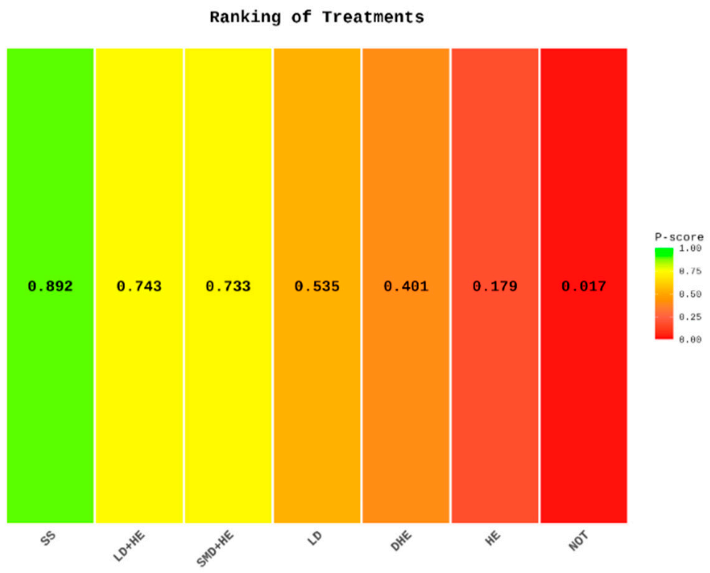

SS (Salt substitutes), LD (Low-sodium diets), SMD (Self-monitoring devices),

DHE (Digital health education), HE (Conventional health education), NOT (No interventions)

P-scores reflect the relative ranking of each intervention in the network meta-analysis, with higher values indicating a greater likelihood of being among the most effective treatment. Salt substitutes (SS) achieved the highest P-score (0.810), followed by low-sodium diet plus health education (LD+HE), self-monitoring devices plus health education (SMD+HE), low-sodium diets (LD), digital health education (DHE), conventional health education (HE), and no intervention (NOT). The colour gradient corresponds to the P-score scale, ranging from red (least effective) to green (most effective).

b) Network of direct comparison among included interventions

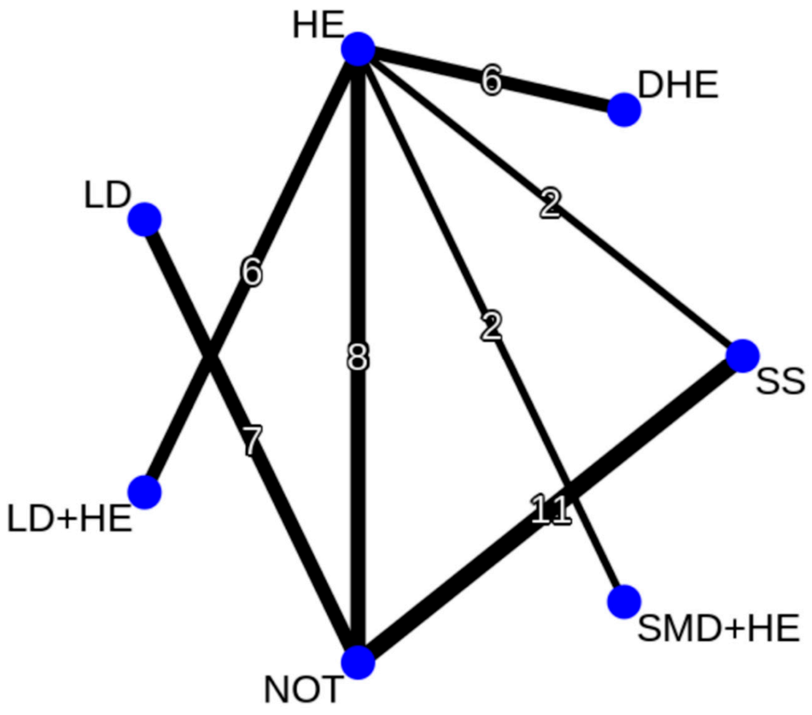

SS (Salt substitutes), LD (Low-sodium diets), SMD (Self-monitoring devices),

DHE (Digital health education), HE (Conventional health education), NOT (No interventions)

Each node represents an intervention, with node size proportional to the total number of participants assigned to that intervention across all trials. Edges indicate direct randomized comparisons between interventions, and the thickness of each edge corresponds to the number of contributing trials. Numerical labels on the lines denote the exact number of studies for each pairwise comparison. SS = salt substitutes; HE = conventional health education; NOT = no intervention; LD = low-sodium diets; LD+HE = low-sodium diets plus health education; SMD+HE = self-monitoring devices plus health education; DHE = digital health education.

c) Mean SBP difference between selected interventions and no intervention (mmHg)

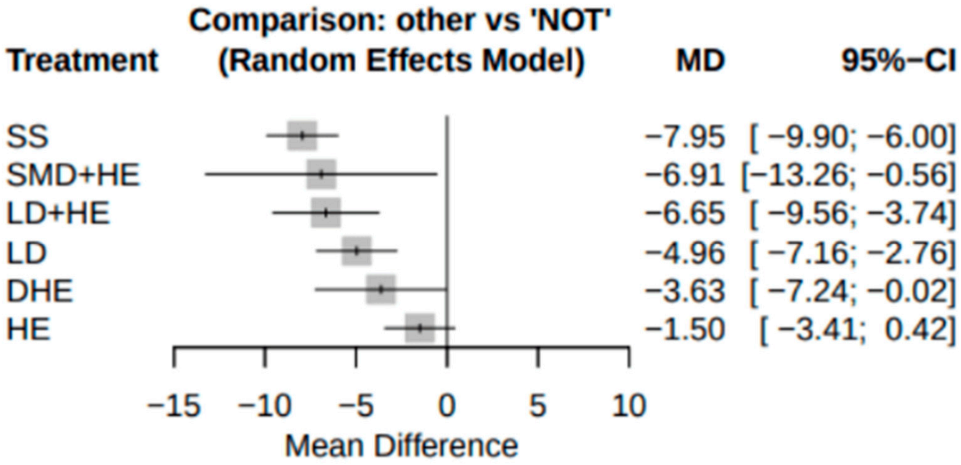

SS (Salt substitutes), LD (Low-sodium diets), SMD (Self-monitoring devices),

DHE (Digital health education), HE (Conventional health education), NOT (No interventions)

Note. The squares presented pooled estimates of the effect size, and the whiskers demonstrated 95% CI). SS (salt substitutes), LD (low-sodium diets), SMD (self-monitoring devices), DHE (digital health education), HE (conventional health education), NOT (no intervention).

c) Mean SBP difference between selected interventions and conventional health education (mmHg)

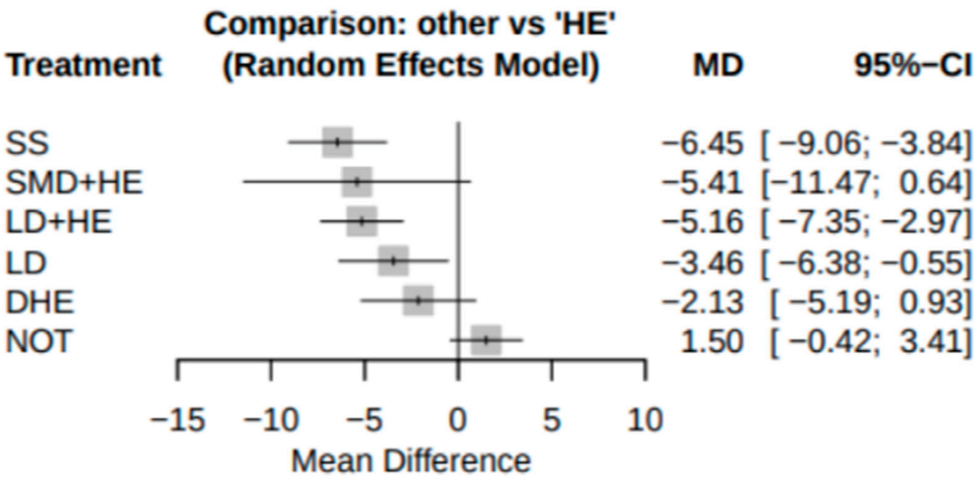

SS (Salt substitutes), LD (Low-sodium diets), SMD (Self-monitoring devices),

DHE (Digital health education), HE (Conventional health education), NOT (No interventions)

Note. The squares presented pooled estimates of the effect size, and the whiskers demonstrated 95% CI). SS (salt substitutes), LD (low-sodium diets), SMD (self-monitoring devices), DHE (digital health education), HE (conventional health education), NOT (no intervention).
